# Pancreatic β cells control glucose homeostasis via the secretion of exosomal miR‐29 family

**DOI:** 10.1002/jev2.12055

**Published:** 2021-01-21

**Authors:** Jing Li, Yujing Zhang, Yangyang Ye, Dameng Li, Yuchen Liu, Eunyoung Lee, Mingliang Zhang, Xin Dai, Xiang Zhang, Shibei Wang, Junfeng Zhang, Weiping Jia, Ke Zen, Antonio Vidal‐Puig, Xiaohong Jiang, Chen‐Yu Zhang

**Affiliations:** ^1^ Nanjing Drum Tower Hospital Center of Molecular Diagnostic and Therapy Chinese Academy of Medical Sciences Research Unit of Extracellular RNA State Key Laboratory of Pharmaceutical Biotechnology Jiangsu Engineering Research Center for MicroRNA Biology and Biotechnology NJU Advanced Institute of Life Sciences (NAILS) Institute of Artificial Intelligence Biomedicine School of Life Sciences Nanjing University Nanjing Jiangsu China; ^2^ Department of Medical Physiology Graduate School of Medicine Chiba University Chiba Japan; ^3^ Wellcome‐MRC Institute of Metabolic Science Addenbrooke's Hospital University of Cambridge Metabolic Research Laboratories Cambridge UK; ^4^ Department of Endocrinology & Metabolism Shanghai Jiao Tong University Affiliated Sixth People's Hospital Shanghai Diabetes Institute Shanghai China; ^5^ Department of Gastroenterology Ruijin Hospital Shanghai Jiaotong University School of Medicine Shanghai China; ^6^ Wellcome Sanger Institute Cambridge UK; ^7^ Cambridge University Nanjing Centre of Technology and Innovation Nanjing China

**Keywords:** exosomal miRNAs, glucose homeostasis, pancreatic β cell‐released miRNAs

## Abstract

Secreted microRNAs (miRNAs) are novel endocrine factors that play essential pathological and physiological roles. Here, we report that pancreatic β cell‐released exosomal miR‐29 family members (miR‐29s) regulate hepatic insulin sensitivity and control glucose homeostasis. Cultured pancreatic islets were shown to secrete miR‐29s in response to high levels of free fatty acids (FFAs) in vitro. In vivo, high levels of FFAs, promoted by either high‐fat diet (HFD) feeding (physiopathological) or fasting (physiological), increased the secretion of miR‐29s into plasma. Intravenous administration of exosomal miR‐29s attenuated insulin sensitivity. The overexpression of miR‐29s in the β cells of transgenic (TG) mice promoted the secretion of miR‐29s and inhibited the insulin‐mediated suppression of glucose output in the liver. We used selective overexpression of traceable heterogenous mutant miR‐29s in β cells to confirm that islet‐derived exosomal miR‐29s target insulin signalling in the liver and blunt hepatic insulin sensitivity. Moreover, in vivo disruption of miR‐29s expression in β cells reversed HFD‐induced insulin resistance. In vitro experiments demonstrated that isolated exosomes enriched in miR‐29s inhibited insulin signalling in the liver and increased hepatic glucose production. These results unveil a novel β cell‐derived secretory signal—exosomal miR‐29s—and provide insight into the roles of miR‐29s in manipulating glucose homeostasis.

## 1 INTRODUCTION

Exosomal microRNAs (miRNAs) have been proposed to be an alternative form of cell‐to‐cell communication and have received increasing attention over the past decade. miRNAs, a class of single‐stranded non‐coding RNAs consisting of 19–22 nucleotides, negatively regulate gene expression at the posttranscriptional level (Bartel, 2004). The canonical wisdom is that miRNAs are generated and exert their function intracellularly. However, mounting evidence shows that miRNAs in cells can be packaged in exosomes and released into the circulation (Valadi et al., 2007; Zhang et al., 2010). Rather than being passively released, exosomal miRNAs, like other cytokines and factors, are selectively secreted in response to specific stimuli (Chen et al., 2012; Zhang et al., 2010), indicating that the secretion of miRNAs is likely related to a particular physiological or pathological process in vivo. Exosomes, a type of extracellular vesicle (EV), are double‐membrane‐bound vesicles with a diameter of 30–150 nm (Raposo & Stoorvogel, 2013). Exosomal miRNAs can be transferred into recipient cells through exosome trafficking. Inside the cell, these miRNAs modulate the expression of target genes and regulate biological functions (Skog et al., 2008; Zhang et al., 2010). Exosomal miRNAs participate in diverse pathological processes, including tumour angiogenesis (Li et al., 2013; Zhuang et al., 2012), immune escape in cancer (Yin et al., 2014), atherosclerosis (Hergenreider et al., 2012), and metabolic disorders (Ji & Guo, 2019). The past decade has witnessed growing interest in exosomal miRNA‐mediated crosstalk between metabolic organs, in which exosomal miRNAs have become endocrine factors that facilitate the network regulation of metabolic homeostasis (Huang‐Doran et al., 2017; Samuelson & Vidal‐Puig, 2018). Several exosomal miRNA‐mediated inter‐organ axes have been elucidated; these axes include adipose tissue‐skeletal muscle crosstalk mediated by miR‐27a (Yu et al., 2018)/miR‐130b (Wang et al., 2013)/miR‐155 (Ying et al., 2017), crosstalk between brown adipose tissue and the liver through exosomal miR‐99b (Thomou et al., 2017), and crosstalk between adipose tissue and the cardiovascular system via miR‐194 (Nie et al., 2018)/miR‐410‐5p (Zou et al., 2018). Nevertheless, the functional roles of exosomal miRNAs are still far from well understood. Thus, exosomal miRNAs released from other organs and their functional roles in recipient organs are worthy of further study.

The pancreas is an endocrine organ that plays a pivotal role in regulating glucose homeostasis. The endocrine cells in the pancreas are clustered and form islets of Langerhans, which maintain glucose homeostasis through releasing various hormones (Chandra & Liddle, 2009). Insulin and glucagon, released by islet β cells and α cells, respectively, accomplish glucose homeostasis by their opposing yet balanced actions in other peripheral organs, including the liver, adipose tissue and skeletal muscle (Goke, 2008). Thus, interplay between the pancreas and other organs forms a regulatory network that modulates glucose homeostasis through secretory factors. Given that exosomal miRNAs act as endocrine factors, we investigated whether pancreatic islet‐secreted exosomes/exosomal miRNAs would communicate with the pancreas and peripheral organs. We selected high levels of free fatty acids (FFAs) as a stimulus, given their regulatory role in glucose homeostasis. First, FFAs modulate insulin release under both physiological and pathological conditions by binding and interacting with G‐protein‐coupled receptor (GPCR) in β cells (Fujiwara et al., 2005). Second, a change in the levels of FFAs causes significant alterations in the metabolic profile. Elevated plasma FFA levels enhanced gluconeogenesis in both healthy subjects and patients with T2D (Chen et al., 1999). Pathological obesity‐associated FFAs contribute to hepatic insulin resistance by inhibiting insulin‐mediated suppressive effects on gluconeogenesis. Conversely, decreasing FFA concentrations in T2D patients or obese subjects improved insulin sensitivity and decreased endogenous glucose production (EGP) (Boden et al., 2002; Boden, 2003). Thus, our study sought to investigate the secretion of exosomal miRNAs from pancreatic islets in response to high levels of FFAs and the functional roles of exosomal miRNAs in glucose homeostasis.

## 2 MATERIALS AND METHODS

### 2.1 Cells, reagents, and antibodies

The MIN 6 mouse pancreatic β cell line was a gift from Dr. Xiao Han at Nanjing Medical University (Nanjing, China). MIN 6 cells were cultured at 37°C in a humidified 5% CO_2_ incubator in RPMI 1640 medium supplemented with 10% foetal bovine serum (FBS), 15 mM HEPES, 1% β‐mercaptoethanol, 100 units/ml penicillin, and 100 μg/ml streptomycin (all Gibco, CA, Carlsbad, USA). The C2C12 mouse myoblast cell line was purchased from the Wuhan Cell Line Collection Centre (Wuhan, China). C2C12 cells were cultured in Dulbecco's modified Eagle's medium (DMEM, Gibco) supplemented with 10% FBS (Gibco), 100 units/ml penicillin (Gibco), and 100 μg/ml streptomycin (Gibco), and differentiation was induced by culturing the cells for 2 days in DMEM (Gibco) containing 2% horse serum (Gibco). The 3T3‐L1 mouse adipocyte cell line was purchased from the Institute of Biochemistry and Cell Biology at the Shanghai Institute for Biological Science at the Chinese Academy of Science (Shanghai, China). 3T3‐L1 cells were maintained in DMEM (Gibco) supplemented with 10% FBS (Gibco), 100 units/ml penicillin (Gibco), and 100 μg/ml streptomycin (Gibco). To induce differentiation, 3T3‐L1 cells were first cultured in DMEM (Gibco) containing 0.5 mM 3‐isobutyl‐1‐methylxanthine (Sigma‐Aldrich, St. Louis, MO, USA), 1 M dexamethasone (Sigma‐Aldrich), 10 mg/l insulin (Sigma‐Aldrich) and 10% FBS (Gibco). After 2 days, the medium was replaced with DMEM (Gibco) supplemented with 10% FBS (Gibco), 100 units/ml penicillin (Gibco), and 100 μg/ml streptomycin (Gibco), and the medium was exchanged every 2 days. After 6 days, the induction of 3T3‐L1 cells was considered complete. Short tandem repeat (STR) profiling and the detection of mycoplasma contamination were performed to authenticate all cell lines. Antibodies against the PI3 kinase p85 were purchased from Cell Signalling Technology (Danvers, MA, USA). GAPDH‐, Ago2‐, CD63‐, CD9‐, TSG101, ALIX, and β‐actin‐specific antibodies were purchased from Santa Cruz Biotechnology (Santa Cruz, CA, USA). Antibodies specific for AKT, pAKT, glycogen synthase kinase (GSK) and pGSK were purchased from Cell Signalling Technology (Danvers). The following synthetic RNA molecules were purchased from Ambion (Austin, TX, USA): mimic‐miR‐29a/b/c, anti‐miR‐29a/b/c and scrambled negative control oligonucleotides (mimic‐ncRNA and anti‐ncRNA).

### 2.2 Animal studies

All animal experimental procedures were conducted in accordance with the National Institutes of Health Guide for the Care and Use of Laboratory Animals and had been approved by the Animal Care Committee of Nanjing University (Nanjing, China). C57BL/6J mice and leptin‐mutant (*Lep^ob/ob^*) mice were purchased from the Model Animal Research Centre of Nanjing University (Nanjing, China). All experimental animals were housed in groups of five per cage on a 12‐h light/dark cycle in a specific pathogen‐free facility at Nanjing University. Diet‐induced obesity was achieved by feeding the animals a high‐fat diet (HFD) for 6 weeks. The HFD consisted of 20% carbohydrates, 20% protein and 60% fat. Intravenous injection was used to administer exosomes or control to wild‐type mice. A total of 50 μg of exosomes diluted in 200 μl of saline was injected into each mouse for a total of four injections per mouse. The same volume of saline was administered as a control. miR‐29s transgenic (TG) mice at 6 weeks of age were maintained on a chow diet since the overexpression of miR‐29s in pancreatic islets was a substitute for the HFD‐induced increase in miR‐29s. Six‐week‐old miR‐29s sponge mice were maintained on the HFD or chow diet accordingly.

### 2.3 Plasma preparation

Thirteen patients with type 2 diabetes (T2D) (nine males and four females, BMI > 25) and eight healthy Chinese donors were recruited from Shanghai Ruijin Hospital. Venous blood samples (∼5 ml) were collected from each donor and placed into 1.5‐ml Eppendorf tubes containing 3.8% sodium citrate. The plasma was fractioned by centrifugation at 3,000 rpm for 15 min at 4°C. The supernatants were recovered and stored at −80°C until further analysis.

### 2.4 Primary pancreatic islet isolation

Pancreatic islets were isolated using collagenase type V digestion as previously described (Cawthorn & Chan, 1991; Zhang et al., 2001). Briefly, 12‐week‐old C57BL/6J mice were anaesthetized, and their pancreatic ducts were cannulated and injected with 1 mg/ml type V collagenase (Sigma‐Aldrich, St. Louis, MO, USA) in D‐Hank's buffered saline solution to inflate the pancreases. The pancreases were then carefully isolated and loaded into digestion solution containing 1 mg/ml type V collagenase. After digestion at 37°C for approximately 28 min, the islets were dispersed via manual agitation. The digested tissue was resuspended in 5 ml of Histopaque (Sigma‐Aldrich, St. Louis, MO, USA) that was then overlaid with 5 ml of D‐Hank's solution. After gradient centrifugation, the islets were collected and transferred to RPMI 1640 medium supplemented with 10% FBS. The islets were cultured at 37°C in a humidified atmosphere consisting of 5% CO_2_/95% air for 12 h (primary culture) to remove exocrine and other tissues. A total of 1200 islets isolated from 10–12 mice were cultured and treated with or without FFAs. After 24 h, pancreatic islets and their conditioned media were collected for RNA analysis or exosome isolation.

### 2.5 Primary hepatocyte isolation

Primary hepatocytes were isolated from C57BL/6J mice as previously described (Castell & Gomez‐Lechon, 2009). Their livers were first perfused with perfusion buffer (Hank's balanced saline solution, HBSS, Gibco) and then with type IV collagenase solution (Gibco). The dispersed cells were resuspended and seeded onto 9.6‐cm^2^ plates in DMEM supplemented with 10% FBS.

### 2.6 Cell co‐culture and exosome incubation assay

A total of 10^4^ primary hepatocytes were plated in the lower chambers of a 6‐well plate, and 200 primary pancreatic islets treated with or without FFAs were placed into the upper chambers. These cells were co‐cultured in the same culture medium. The underneath surface of the upper chamber contained a 0.5‐μm Transwell polycarbonate membrane through which the exosomes, but not the cells, could pass. The co‐cultured cells were therefore able to exchange signalling molecules. For the exosome incubation assay, 30 μg of exosomes was incubated with hepatocytes. After 6 h, hepatocytes were harvested for miR‐29 expression assays or the hepatic glucose output assay. Exosome‐free FBS was prepared and used in all co‐culture and exosome incubation experiments.

### 2.7 Transfection of cells with ncRNA, mimic‐miR‐29 or anti‐miR‐29

MIN 6 cells were seeded into 75‐cm^2^ flasks overnight and transfected the next day using Lipofectamine 2000 (Invitrogen, Carlsbad, CA, USA) according to the manufacturer's instructions. Mimic‐miR‐29a/b/c (pre‐miR‐29a/b/c), anti‐miR‐29a/b/c, scrambled mimic‐miR‐29 (mimic‐ncRNA), and scrambled anti‐miRNA (anti‐ncRNA) were purchased from Ambion (USA). To increase the expression of miR‐29 family members, 600 pmol of mimic‐miR‐29s (200 pmol of each family member) was mixed together and transfected into 1×10^7^ MIN 6 cells. Scrambled mimic‐miRNA (200 pmol) was used as a negative control. To knock down miR‐29 expression in MIN 6 cells, the same amount of anti‐miR‐29 was used, and scrambled anti‐miRNA was used as a negative control. 6 h later, the spent Opti‐MEM was replaced with fresh RPMI 1640 culture medium; 24 h later, the culture medium was collected and prepared for exosome isolation.

### 2.8 Exosome isolation

Exosomes were isolated from human plasma or cell culture medium via differential centrifugation as previously described (Thery et al., 2006; Valadi et al., 2007). Briefly, the culture medium was centrifuged at 300 × *g* for 5 min and then at 3000 × *g* for 20 min to remove the cells and other debris, followed by centrifugation at 10,000 × *g* for 30 min to remove large vesicles. Then, the supernatant was centrifuged at 110,000 × *g* for 70 min. Exosomes were collected from the pellet and resuspended in FBS‐free medium or saline. All centrifugation steps were performed at 4°C. For each replicate experiment, 1,200 islets, 4 × 10^6^ primary hepatocytes, and 10^7^ MIN 6/C2C12/3T3‐L1 cells were cultured and treated with FFAs for 24 h. Then, the culture medium was collected to isolate exosomes, which were then used to analyse the secretion of miR‐29s under high‐FFA conditions. For tail vein injections, the cultured medium from 0.6×10^7^ MIN 6 cells was harvested and used to inject each mouse.

### 2.9 Transmission electron microscopy (TEM)

Exosome pellets were first fixed overnight at 4°C in a droplet of 2.5% glutaraldehyde in PBS buffer at pH 7.2. Then, the samples were washed with PBS three times (10 min each) and post‐fixed in 1% osmium tetroxide for 60 min at room temperature. Then, samples were embedded in 10% gelatine, fixed in glutaraldehyde at 4°C and cut into several blocks (less than 1 mm^3^ in volume), followed by a 10‐min dehydration steps in alcohol at increasing concentrations (30%, 50%, 70%, 90%, 95%, and 100% ×3). Pure alcohol was then replaced with propylene oxide, and the samples were infiltrated with Quetol 812 epoxy resin at increasing concentrations (25%, 50%, 75%, and 100%) with propylene oxide for a minimum of 3 h per step. Next, the samples were embedded in 100% fresh Quetol 812 and polymerized at 35°C for 12 h and then 60°C for 24 h. Ultrathin sections (100 nm) were obtained from the prepared samples using a Leica UC6 ultra‐microtome. Finally, the samples were post‐stained with uranyl acetate for 10 min and with lead citrate for 5 min at room temperature and observed in an FEI Tecnai T20 transmission electron microscope operated at 120 kV.

### 2.10 Nanoparticle tracking analysis (NTA)

Exosomes were first resuspended in saline at a concentration of 5 μg of protein/ml and further diluted from 100‐ to 500‐fold for analysis. NTA was performed using the NanoSight NS300 system (NanoSight), which focuses a laser beam through a suspension of the particle of interest. The results were visualized by light scattering.

### 2.11 Generation of fluorescently labelled exosomes

CD63 cDNA was amplified and cloned into the pEGFP‐N1 vector (Addgene, MA, USA) as described previously (Ye et al., 2020). Then, plasmid for CD63‐EGFP expression was transfected into cells. After 24 h, exosomes were isolated from the conditioned medium and intravenously administered to mice.

### 2.12 Confocal microscopy analysis of fluorescently labelled miRNAs

MIN 6 cells were transfected with fluorescently labelled miR‐29a (Invitrogen). Next, exosomes were isolated from the MIN 6 cell culture medium as previously described. The MVs were incubated with cultured hepatocytes for 6 h in RPMI 1640 medium supplemented with 10% FBS. The hepatocytes were washed, fixed and then observed using a confocal microscope (FV1000; Olympus, Tokyo). The excitation wavelengths were 405 nm for DAPI and 532 nm for fluorescently labelled miR‐29a. The image size was 1024×1024 pixels.

### 2.13 RNA isolation and quantitative RT‐PCR of mature miRNAs

Total RNA was isolated from exosomes, cells, plasma and tissues using TRIzol reagent (Invitrogen, Carlsbad, CA, USA) according to the manufacturer's instructions. Quantitative RT‐PCR was performed using TaqMan miRNA probes (Applied Biosystems, Foster City, CA, USA) according to the manufacturer's instructions. Briefly, cDNA was synthesized from 5 μl of total RNA using AMV reverse transcriptase (TaKaRa, Dalian, China) and a stem‐loop RT primer (Applied Biosystems). Real‐time PCR was performed using a TaqMan PCR kit on an Applied Biosystems 7300 sequence detection system (Applied Biosystems). All reactions, including the no‐template control reactions, were run in triplicate. C_T_ values were then determined using fixed threshold settings. To calculate the absolute expression levels of target miRNAs, a series of synthetic miRNA oligonucleotides at known concentrations were also reverse‐transcribed and amplified. The absolute amount of each miRNA was then calculated on the basis of a standard curve. Expression of the miRNAs in the cells was normalized to the level of U6 small nuclear RNA (snRNA).

### 2.14 Western blot analysis

Whole‐cell lysates used for western blot analysis were extracted using RIPA buffer (Biyuntian, Nanjing, China). The cell lysates were loaded onto SDS‐PAGE gels and transferred to polyvinylidene fluoride membranes. Protein expression was determined using an enhanced chemiluminescence western blot detection kit (Amersham Biosciences). Ponceau staining of the uncropped membrane was used as a loading control. Normalisation was performed after the same samples were blotted with antibody against α‐tubulin, AKT or GSK accordingly.

### 2.15 Oil red O staining

Oil Red O staining was performed as previously described (Zheng et al., 2019). Briefly, livers were fixed in 4% paraformaldehyde for 24 h, embedded in OCT compound, and sectioned using a microtome. The slides were rinsed with 70% ethanol for 2 min, followed by the use of oil Red O (Sigma, Deisenhogfen, Germany) to stain the lipids for 15 min. The slides were then rinsed with 75% ethanol for 5 min, washed with PBS and imaged immediately.

### 2.16 Biotin‐miRNA pull‐down assay

3′‐Biotinylated miR‐29a and biotinylated scrambled oligonucleotide were synthesized from Genscript (Nanjing, China) and transfected into mouse hepatoma cells (Hepa1‐6). After 24 h, the cells were lysed, and the lysates were incubated with streptavidin magnetic beads (Thermo Fisher Scientific, MA, USA) for 4 h. The beads were collected by centrifugation at 2500 rpm for 5 min at 4°C. Immunoprecipitants were eluted from the beads for mRNA analysis.

### 2.17 In vivo and in vitro insulin signalling

Following a 16‐h fast, the mice were intraperitoneally injected with 1 U/kg insulin. Their livers were removed and frozen in liquid nitrogen for 10 min following insulin injection. Then, the proteins were extracted for western blot analysis. For in vitro analyses, the cultured hepatocyte medium was removed, and the cells were washed twice. Fresh medium supplemented with 10% FBS was added, and 0.2 nM human insulin was used to stimulate the hepatocytes for 10 min. The cells were harvested, and the proteins were then extracted for western blot analysis.

### 2.18 Hepatic glucose production assay

Hepatic gluconeogenesis was quantified using a glucose assay kit purchased from Sigma‐Aldrich (MO, USA). Hepatocytes were first treated with exosomes for 6 h. Then, the culture medium was replaced with HBSS supplemented with 100 nM insulin. Twenty minutes later, the supernatant was collected, and the assay was performed according to the manufacturer's instructions.

### 2.19 Plasmid construction and generation of a TG mouse model

The pcDNA6.2‐GW/EmGFP‐miR (Invitrogen, Carlsbad, CA, USA) construct was used to generate all three types of TG mice. To achieve islet‐specific expression of the inserted cassette, the CMV promoter controlling miR‐29s and GFP was replaced with the rat insulin 2 promoter RIP2.

For the miR‐29s TG plasmid, mimic precursors of miR‐29a, miR‐29b, and miR‐29c connected were cloned in series into the vector and co‐expressed with GFP under the control of RIP2. For the miR‐29a^mut^ plasmid, mutant miR‐29a was designed and synthesized by Invitrogen (USA). The sequence of the mutant miR‐29a, in which four nucleotides in a non‐seed region were mutated, was 5ʹ‐uagcaccaucagacaucaguaa‐3ʹ. Then, the GFP sequence was removed from the vector, and the mutant miR‐29a precursor sequence was cloned into the same vector under the control of RIP2. To increase the expression efficiency, three copies of mutant miR‐29a were inserted into the vector. For the miR‐29s^def^ plasmid, a sequence encoding a string of specific antisense sequences to miR‐29s (sponge) was designed as proposed by a previous study (Ma et al., 2011). The sponge construct contained three copies of an antisense sequence for each miR‐29 member. Then, the sponge construct was cloned into the same vector and co‐expressed with GFP under the same promoter control. Blastocyst injection was performed at the Model Animal Research Centre of Nanjing University. For all types of TG mice, founder mice were hybridized with wild‐type C57BL/6J mice to produce offspring. Genomic DNA was identified by PCR assay.

### 2.20 Glucose‐stimulated insulin secretion (GSIS)

Various mouse pancreatic islets were incubated in Krebs‐Ringer bicarbonate buffer supplemented with 0.1% BSA. After preincubation for 15 min, fractions of the perfusate were collected at 1‐min intervals. Then, the glucose concentration in the perfusion medium was changed to 16.7 mM. Insulin was measured by a radioimmunoassay kit (Linco/Millipore, St. Charles, MO) (Ainscow et al., 2000).

### 2.21 Insulin tolerance test (ITT) and glucose tolerance test (GTT)

The ITT was performed in mice after a 6‐h fast. After fasting, blood glucose levels were determined, and the animals received an intraperitoneal dose of 0.75 U/kg body weight insulin. Blood glucose levels were determined after 15, 30, 45, 60, and 90 min. GTTs were performed after a 16‐h overnight fast. After basal blood glucose levels had been measured, each animal was treated with 2 g/kg body weight glucose. Blood glucose levels were recorded after 15, 30, 45, 60, and 90 min.

### 2.22 Hyperinsulinaemic‐euglycaemic clamp studies

Hyperinsulinaemic‐euglycaemic clamp analyses were conducted as previously described (Kornfeld et al., 2013; Qiu et al., 2014). Briefly, catheters were surgically implanted into the right jugular vein at least 5 days before hyperinsulinaemic‐euglycaemic clamping was performed. After 5 days of recovery, each animal was fasted for 5 h and then placed in individual restraints to cut tail samples. A 5‐μCi bolus of [3‐^3^H] glucose (PerkinElmer) was administered starting at 90 min prior to clamping and followed by infusion of 0.05 μCi/min for a 90‐min equilibration period. After a 90‐min basal period, a blood sample was collected from the tail tip to determine the basal parameters. Clamping was initiated with primed (300 mU/kg) and continuous (2.5 mU/kg/min) insulin infusion, and the [3‐^3^H] glucose infusion rate was increased to 0.1 μCi/min for the remainder of the clamping period. Physiological blood glucose levels (∼120–130 mg/dl) were maintained by adjusting a 20% glucose infusion. Blood samples were collected every 10 min. Approximately 120 min before the end of the experiment, [3‐^3^H] glucose was infused, and blood samples were collected until a steady state was achieved. EGP and whole‐body glucose clearance were determined as previously described.

### 2.23 Statistical analyses

Statistical analyses were performed using GraphPad Prism 8.0. Comparisons between two groups were performed with the two‐tailed unpaired Student's t‐test or analysis of variance (ANOVA). Western blots images are representative of at least three independent experiments. qRT‐PCR was performed in triplicate, and each experiment was repeated several times. Differences were considered statistically significant at *P* < 0.05. Data are presented as the mean ± standard error of the mean (SEM).

## 3 RESULTS

### 3.1 Cultured pancreatic islets secreted miRNAs in response to FFA treatment

**FIGURE 1:**
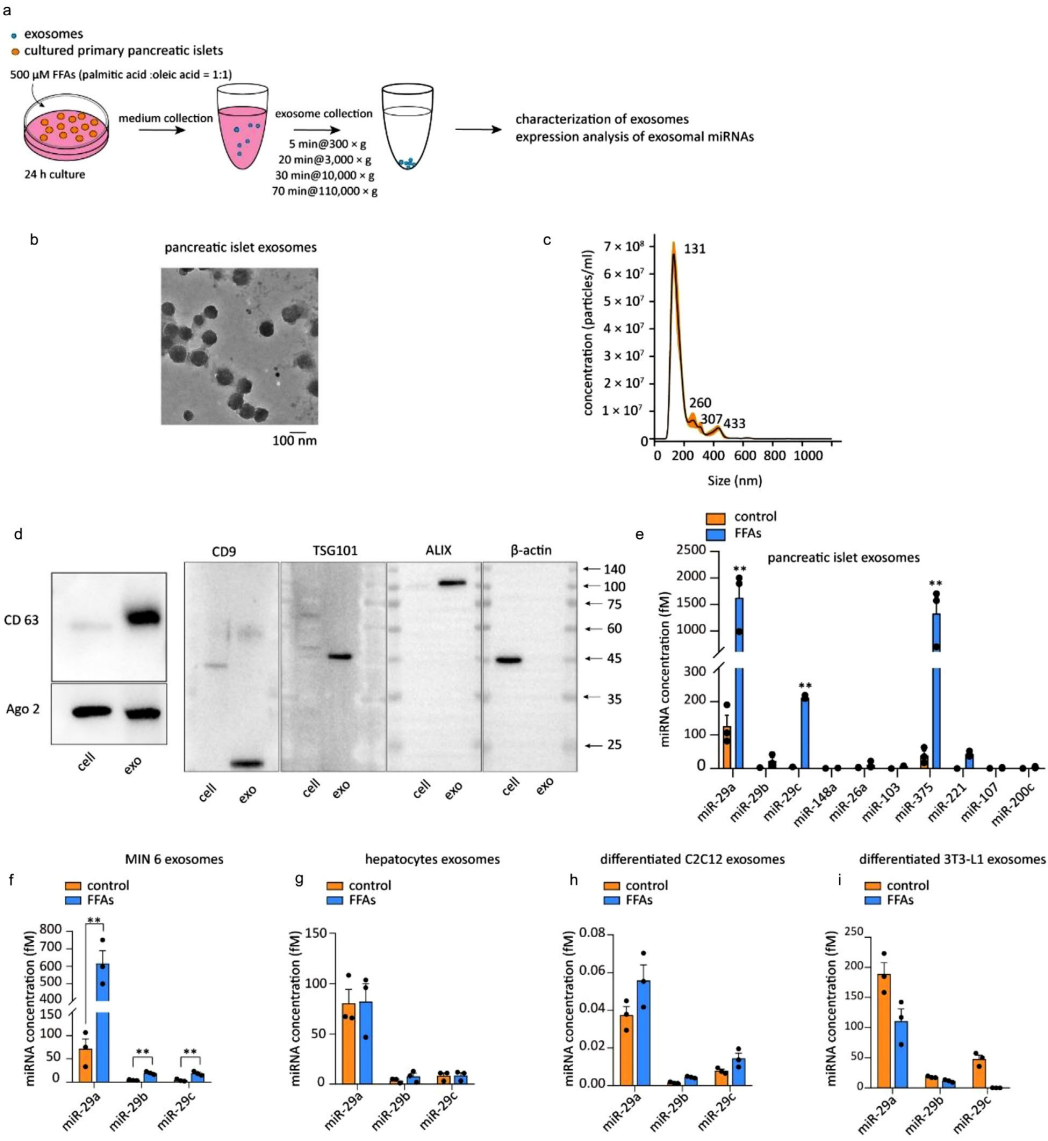
(a) Schematic of the isolation of exosomes from pancreatic islets. (b) TEM image of exosomes isolated from the conditioned medium of pancreatic islets after FFA treatment for 24 h. (c) Nanoparticle tracking analysis (NTA) of exosomes isolated from the conditioned medium of pancreatic islets after FFA treatment for 24 h. (d) Representative western blot of the exosomal membrane markers CD63 and CD9 and proteins that mediate the budding and abscission processes during exosome exocytosis in FFA‐treated islets and their exosomes. (e) qPCR analysis of the miRNA concentrations in exosomes isolated from the conditioned medium of pancreatic islets after FFA treatment for 24 h. Islets from ten mice were pooled and cultured together; these islets represent each spot in the figure. (f‐i) qPCR analysis of the miR‐29s concentrations in exosomes isolated from the conditioned medium of MIN 6 cells (f), primary hepatocytes (g), differentiated C2C12 cells (h) and differentiated 3T3‐L1 cells (I) after FFA treatment for 24 h

Mouse primary pancreatic islets were isolated and cultured in vitro to investigate the secretion of exosomes (Figure S1A). FFAs (palmitic acid:oleic acid at 1:1, 500 μM) were applied to primary pancreatic islets for 24 h (Pusl et al., 2008) to mimic hyperlipidaemic conditions in vitro. Exosomes were collected from conditioned medium using ultracentrifugation (Figure 1a) and identified by TEM, NTA and specific biomarker assessment. The exosomes, which presented diameters of approximately 100 nm, were discernible under TEM (Figure 1b,c). Exosomal membrane protein markers (CD63 and CD9) and proteins that mediate the budding and abscission processes during exosome exocytosis (ALIX and TSG101) were distinctively detected in the exosomes (Figure 1d). These results show that pancreatic islets secrete exosomes into the extracellular environment. Next, we profiled the miRNAs in islet‐derived exosomes to identify candidate miRNAs that mediate intercellular crosstalk. The following several reported miRNAs known to be related to glucose metabolism and islet functions were analysed: miR‐29a, miR‐29b, miR‐29c, miR‐148a, miR‐26a, miR‐103, miR‐375, miR‐221, miR‐207, and miR‐200c (Chakraborty et al., 2014; Fu et al., 2015; He et al., 2007). Of all the tested miRNAs, the miR‐29 family members‐miR‐29a/b/c (miR‐29s) attracted our attention due to their significantly increased levels in FFA‐induced islet exosomes (Figure 1e). Moreover, the level of β cell‐specific miR‐375 was also markedly increased in FFA‐induced exosomes (Figure 1e). Given that intracellular miR‐29s contribute to insulin resistance (He et al., 2007; Massart et al., 2017), we wondered about the contribution of islet‐derived exosomal miR‐29s in our study. We found that the levels of miR‐29s in pancreatic islets increased upon treatment with FFAs (Figure S1B). miR‐29s were also secreted from the MIN 6 β cell line upon FFA administration (Figure 1f). FFA‐induced secretion of miR‐29s could be abrogated by the exosome secretion inhibitor GW4869, thus demonstrating that the secretion of miR‐29s is associated with the release of exosomes (Figure S1C). We also investigated the secretion of miR‐29s from hepatocytes, C2C12 skeletal myocytes and 3T3‐L1 white adipocytes. These cell lines were selected because they effectively model the cellular mechanisms controlling glucose homeostasis. Notably, the levels of miR‐29s did not change in any of these cell lines (Figure S1D‐S1F) or in their exosomes upon treatment with FFAs (Figure 1g‐i). Collectively, these results suggest that pancreatic islets secrete exosomal miR‐29s in response to the secretagogue FFAs.

### 3.2 Pancreatic islets secreted miR‐29s in response to FFAs in vivo under physiological and pathological conditions

**FIGURE 2:**
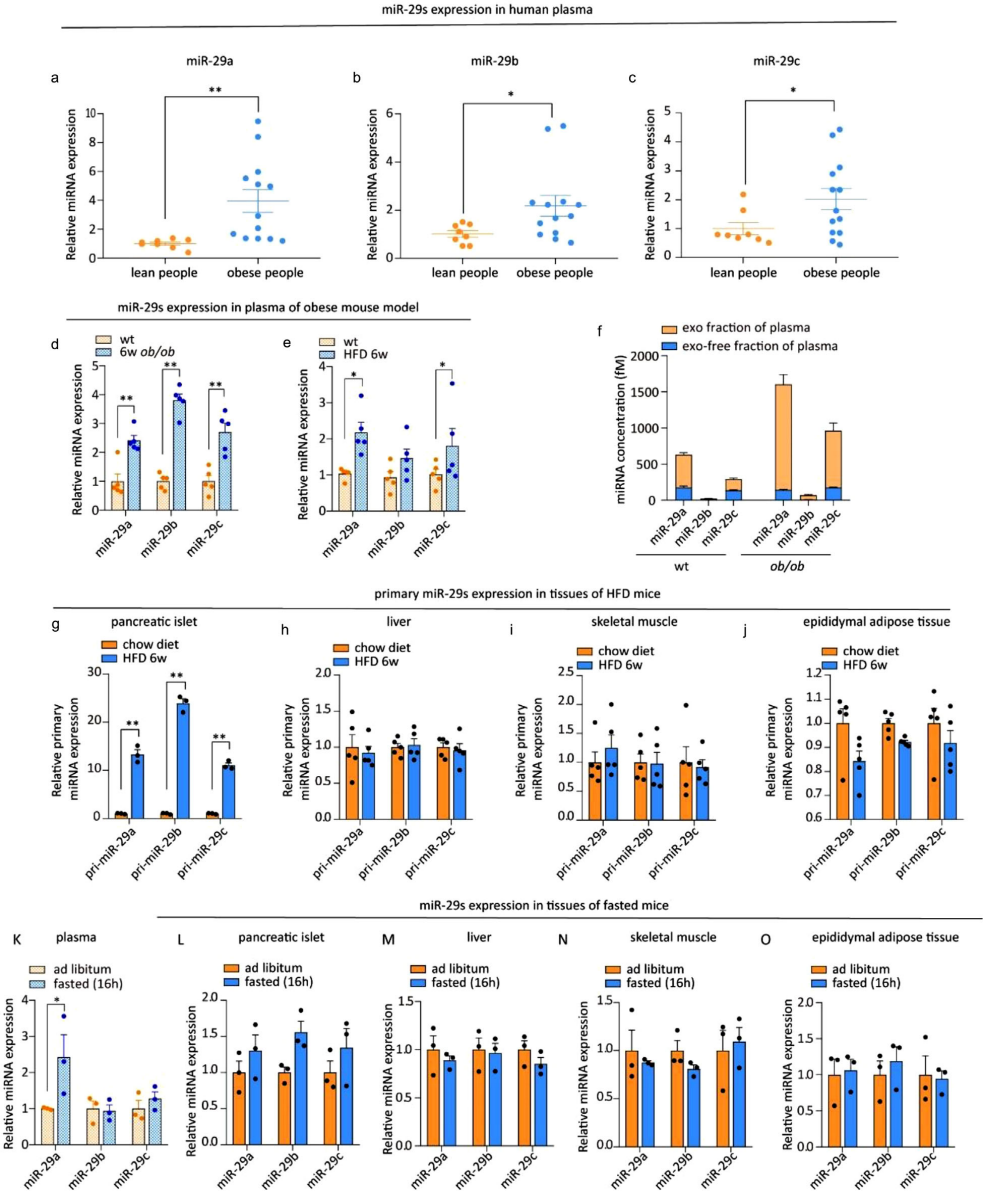
(a‐c) qPCR analysis of the levels of miR‐29a, miR‐29b, and miR‐29c in the plasma of healthy donors (*n* = 8) and obese people (*n* = 13). (d) qPCR analysis of the levels of miR‐29s in the plasma of 6‐week‐old, male, wild‐type and *ob/ob* mice (*n* = 5 for each group). (e) qPCR analysis of the levels of miR‐29s in the plasma of mice fed a chow diet or HFD (*n* = 5 for each group). (f) qPCR analysis of the miR‐29s concentrations in the exosome‐free plasma fraction or exosomal fraction isolated from the plasma of 6‐week‐old, male, wild‐type or *ob/ob* mice. Plasma was obtained from 15 mice in each group and pooled to harvest exosomes. (g‐j) qPCR analysis of the levels of primary miR‐29s in pancreatic islets (g), livers (h), skeletal muscles (i) and gonadal adipose tissues (j). Islets from three mice were pooled together and represent a single spot in panel g (*n* = 5 for each group in panels h, i and j). (k‐o) qPCR analysis of the levels of miR‐29s in the plasma (k), pancreatic islets (l), livers (m), skeletal muscles (n) and gonadal adipose tissues (o) of mice fed an ad libitum diet or fasted for 16 h (*n* = 3 for each group). Islets from three mice were pooled together and represent a single spot in panel L (*n* = 3 for each group in panels l, m, and n)

Next, we investigated whether miR‐29s would be secreted in vivo under high FFAs levels. Elevated plasma FFAs in vivo are associated with obesity. First, we investigated the levels of miR‐29s in the circulation of patients with both obesity (BMI > 25) and T2D diabetes (Table S1). Of relevance, the levels of miR‐29a, miR‐29b and miR‐29c were significantly increased in the plasma of patients with both obesity and diabetes (Figure 2a‐c). Circulating miR‐29s were also substantially increased in obese animals, including *ob/ob* mice (Figure 2d) and HFD‐fed mice (Figure 2e). A large proportion of circulating miRNAs are in cell‐derived exosomes. Thus, the plasma of *ob/ob* mice was fractionated, and miR‐29s levels in both exosomes and the exosome‐free fraction were analysed. TEM and NTA were used to confirm that the isolated vesicles were bona fide exosomes by determining their morphology and size (Figure S2A and S2B). We found that most of the circulating miR‐29s were packaged in exosomes (Figure 2f). Exosomal miR‐29s (the exo fraction of plasma) were significantly increased in the plasma of *ob/ob* mice compared to that of wild‐type mice (Figure 2f). Since primary cultured pancreatic islets and MIN 6 cells secreted miR‐29s in vitro, we next investigated whether these stably expressed exosomal miR‐29s originated from pancreatic islets in vivo. We determined the endogenous transcriptional levels of miR‐29s in pancreatic islets isolated from HFD‐fed mice. Endogenous mature miRNAs are successively processed from primary miRNAs and precursor miRNAs, so the endogenous transcriptional levels of miR‐29s can easily be analysed by measuring primary miRNA levels. We found that the levels of primary miR‐29s were significantly increased in pancreatic islets (Figure 2g). Conversely, primary miR‐29s levels in the liver (Figure 2h), skeletal muscle (Figure 2i) and gonadal adipose tissue (Figure 2j) were not affected. These results were consistent with previous results showing that the levels of miR‐29s were increased exclusively in exosomes derived from FFA‐treated pancreatic islets (Figure 1f‐i). Hyperinsulinaemia is another obesity‐associated condition that may account for increased levels of circulating miR‐29s as well. To investigate this, 0.1 pM insulin (similar to the concentration in HFD‐fed mice) was applied to MIN 6 cells. However, high levels of insulin could not induce the secretion of miR‐29s from MIN 6 cells (Figure S2C), further supporting the notion that miR‐29s are secreted specifically in response to high levels of FFAs. In addition to obesity, fasting is a physiological condition that triggers elevated FFAs in plasma. Upon fasting for 16 h, blood glucose was decreased (Figure S2D), and plasma FFA levels were significantly increased (Figure S2E). Accordingly, the levels of miR‐29s were raised in the plasma of fasted mice compared to that of mice on an ad libitum diet (Figure 2k). Similarly, to investigate the origin of these circulating miR‐29s, the levels of miR‐29s in four essential tissues that manage glucose metabolism were also analysed. The levels of miR‐29s were slightly and mainly increased in the pancreatic islets of fasted mice Figure (2l‐o), indicating that the increased circulating miR‐29s under fasted conditions likely originated from pancreatic islets. Altogether, these results suggest that miR‐29s are secreted extracellularly and circulate in response to high levels of FFAs under both physiological and pathological conditions.

### 3.3 Intravenous administration of pancreatic islet‐secreted miR‐29s attenuated systemic insulin sensitivity

**FIGURE 3:**
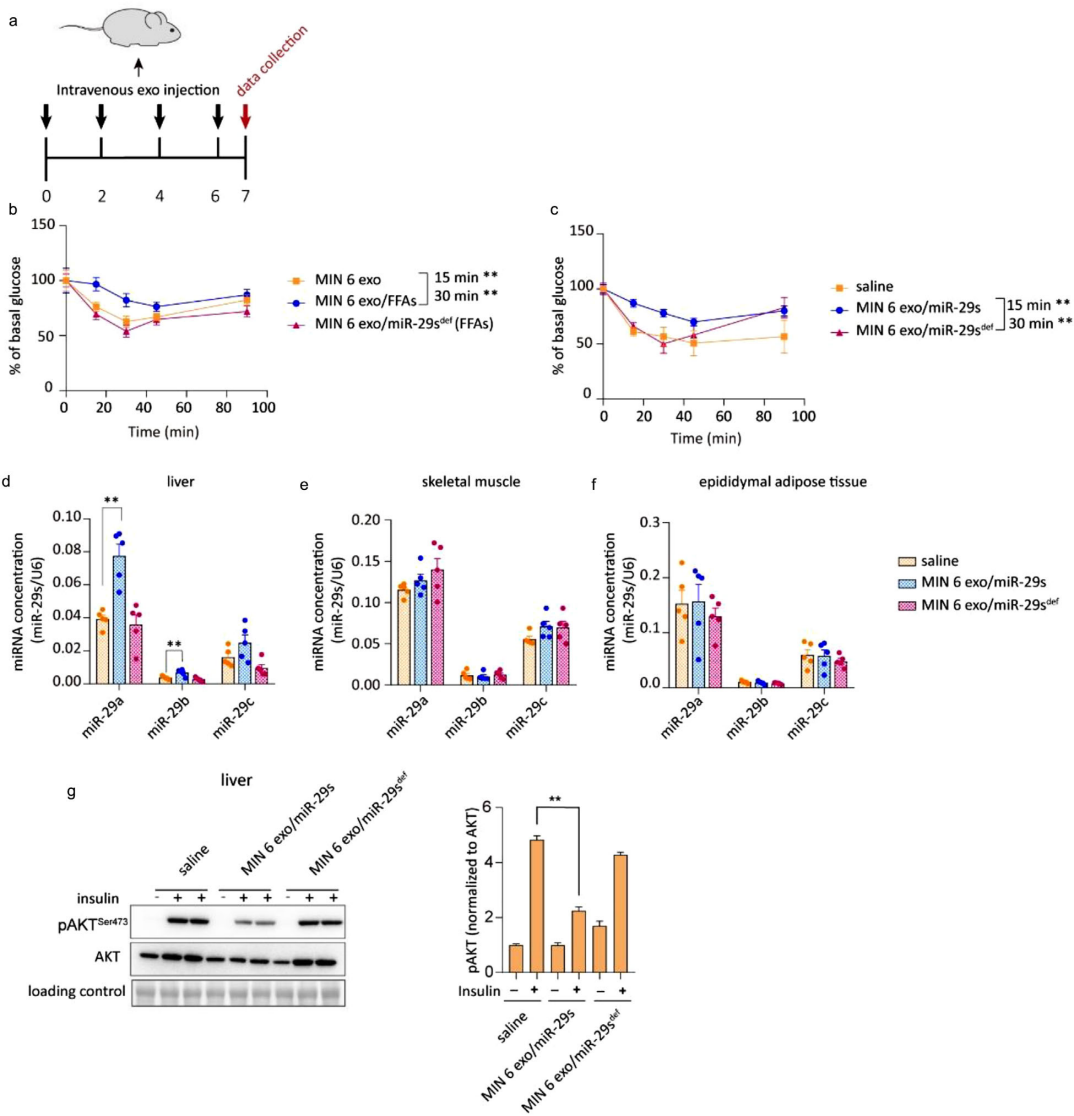
(a) Schematic of the intravenous injection of exosomes. Exosomes were administered once every 2 days for a total of four injections. Fifty micrograms of exosome was injected each time. (b) The insulin tolerance test (ITT) was administered to mice injected with various exosomes as indicated. Wild‐type mice were inoculated intravenously with exosomes isolated from untreated or FFA‐treated MIN 6 cells or MIN 6 cells co‐treated with FFAs and miR‐29s antagomir (*n* = 8 for each group). (c) The insulin tolerance test (ITT) was administered to mice injected with various exosomes as indicated. Wild‐type mice were inoculated intravenously with saline or exosomes isolated from miR‐29s‐overexpressing MIN 6 cells or miR‐29s‐depleted MIN 6 cells (*n* = 8 for each group). (d‐f) qPCR analysis of the levels of miR‐29s in livers (d), skeletal muscles (e) and gonadal adipose tissues (f) (*n* = 5 for each group). (g) Representative western blot of insulin‐stimulated AKT phosphorylation in the livers of mice injected with saline or exosomes replete with or depleted of miR‐29s (left panel). Analysis of western blots from *n* = 3 independent experiments (right panel)

Although local miR‐29s contribute to the regulation of insulin sensitivity (He et al., 2007), the functional role and relevance of miR‐29s secreted from pancreatic islets remain unknown. MIN 6 cell‐derived exosomes depleted of or replete with miR‐29s were intravenously injected into mice, as shown in Figure 3a. Bodyweight, fasting insulin levels and fasting glucose levels were unaltered after injection of the exosomes (Figure S3A‐S3C). Insulin sensitivity was assessed by the intraperitoneal GTT (i.p. GTT) and ITT. Exosomes from FFA‐treated MIN 6 cells containing abundant miR‐29s (Figure S3D) impaired insulin sensitivity (Figure 3b). Conversely, the administration of exosomes produced by FFA‐treated miR‐29s‐deficient MIN 6 cells containing few miR‐29s (Figure S3D) failed to affect insulin sensitivity (Figure 3b). To further confirm that exosomal miR‐29s, but not other exosome components, are the determinants of systemic insulin resistance, we overexpressed and knocked down miR‐29s in exosomes (Figure S3E and S3F). As anticipated, the administration of exosomes overexpressing miR‐29s caused systemic insulin resistance in mice. Conversely, the administration of exosomes depleted of miR‐29s did not affect glucose homeostasis (Figure 3c). To further exclude the possibility that protein cargo in the exosomes impaired insulin sensitivity, we conducted a proteomic analysis of FFA‐induced exosomes. KEGG pathway analysis revealed that the proteins enriched in FFA‐induced exosomes exhibited weak associations with the insulin signalling pathway or glucose metabolism (Figure S3G). Together, these data suggest that high FFA levels increase the secretion of miR‐29s from pancreatic islets and contribute to FFA‐induced insulin resistance.

To further dissect the mechanism underlying miR29‐mediated systemic insulin resistance, we analysed miR‐29s levels and their effects on insulin action in the liver, skeletal muscle and gonadal adipose tissue of mice injected with exosomes. miR‐29s levels were significantly increased in the liver but remained unchanged in skeletal muscle and adipose tissue (Figure 3d,f). This tissue distribution indicated that the liver predominantly took up the injected exosomal miR‐29s. To strengthen this conclusion, we tracked the exosome distribution using engineered fluorescently labelled exosomes. The exosomal membrane protein CD63 was fused with GFP, and a plasmid for the expression of this fusion protein was transfected into cells, as shown in Figure S3H. Confocal images revealed that GFP was discernible in sections of the liver; however, no GFP signal could be observed in the skeletal muscle or adipose tissue (Figure S3I). Next, insulin‐stimulated AKT activation was analysed in these highly metabolic glucose‐regulating tissues. Compatible with the observation that miR‐29s mainly travelled to the liver, mice injected with exosomes containing abundant miR‐29s exhibited decreased insulin‐stimulated AKT activation in the liver (Figures 3g and S4A) but not the skeletal muscle or adipose tissue (Figure S4B and S4C). These results indicate that the role of exosomal miR‐29s in promoting systemic insulin resistance mainly depends on the liver. Notably, although insulin resistance is also closely associated with lipid accumulation, we did not detect enhanced lipogenesis (Figure S4D) or lipid accumulation (Figure S4E) in the livers of mice that intravenously received exosomal miR‐29s. Thus, insulin resistance in this context appears to depend on impaired insulin signalling caused by exosomal miR‐29s.

### 3.4 Overexpression of miR‐29s in pancreatic islets attenuated insulin sensitivity in vivo

**FIGURE 4:**
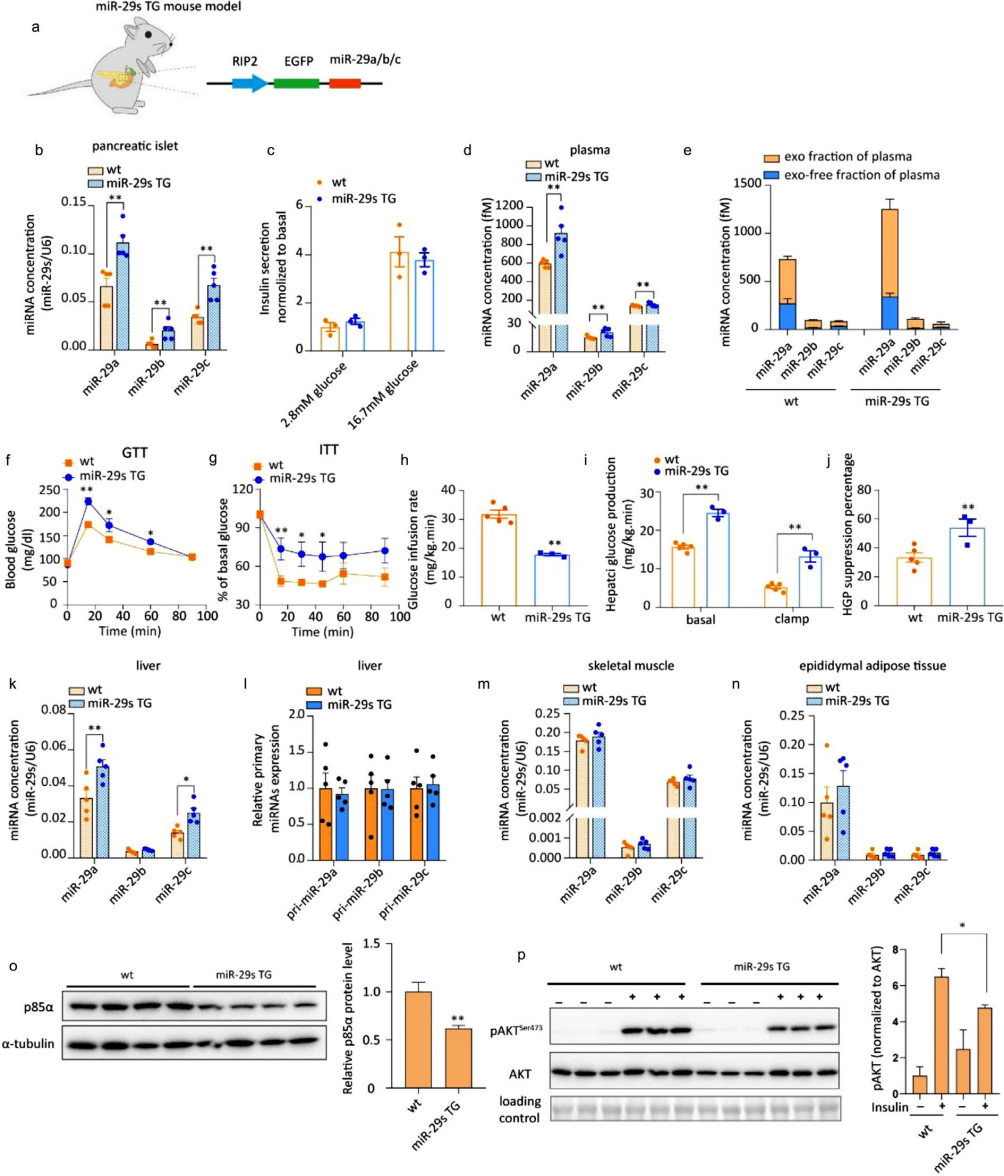
(a) Schematic of the generation of mice whose pancreatic β cells overexpressed miR‐29s. (b) qPCR analysis of miR‐29s concentrations in the pancreatic islets of wild‐type or miR‐29s TG mice. Islets from three mice were pooled together and represent a single spot (*n* = 15 for each group). (c) Glucose‐stimulated insulin secretion (GSIS) of cultured pancreatic islets. Levels of insulin secretion were normalized to the amount of protein in the islets. (d) qPCR analysis of the miR‐29s concentrations in the plasma of wild‐type and miR‐29s TG mice (*n* = 5 for each group). (e) qPCR analysis of the miR‐29s concentrations in the exosome‐free plasma fraction or exosomal fraction isolated from the plasma of wild‐type or miR‐29s TG mice. Plasma was obtained from 15 mice in each group and pooled to harvest exosomes. (f) The intraperitoneal glucose tolerance test (i.p. GTT) was administered to wild‐type or miR‐29s TG mice (*n* = 15 for each group). (g) The insulin tolerance test (ITT) was administered to wild‐type or miR‐29s TG mice (*n* = 8 for each group). (h‐i) Hyperinsulinaemic‐euglycaemic clamping of wild‐type or miR‐29s TG mice. The glucose infusion rate (h) and hepatic glucose production (i) by insulin are expressed as mg/kg.min. The percentage of HGO suppression (j) was analysed according to the results in panel I. (k) qPCR analysis of the miR‐29s concentrations in the livers of wild‐type or miR‐29s TG mice (*n* = 5 for each group). (l) qPCR analysis of the expression of primary miR‐29s levels in the livers of wild‐type or miR‐29s TG mice (*n* = 5 for each group). (m‐n) qPCR analysis of the concentrations of miR‐29s in the skeletal muscle and adipose tissue of wild‐type or miR‐29s TG mice (*n* = 5 for each group). (o) Representative western blot of p85α and α‐tubulin in the livers of wild‐type or miR‐29s TG mice (left panel). Analysis of p85α western blots from *n* = 3 independent experiments (right panel). (p) Representative western blot of insulin‐stimulated AKT phosphorylation in the livers of wild‐type or miR‐29s TG mice (left panel). Analysis of western blots from *n* = 3 independent experiments (right panel)

To further investigate the role of islet‐secreted miR‐29s in glucose homeostasis, we generated a TG mouse line whose pancreatic β cells selectively overexpressed miR‐29s (hereafter referred to as miR‐29s TG mice). To limit miR29s expression to pancreatic β cells, miR‐29a/b/c were driven by the RIP promoter, which is regulated in β cells (Figure 4a). The fluorescent protein GFP under the control of RIP was used as a readout. PCR analysis of GFP expression demonstrated that the insertion cassette was clearly expressed in the pancreatic islets (Figure S5A). Additionally, the results of confocal microscopy confirmed expression of the insertion cassette in isolated pancreatic islets (Figure S5B). There were no overt differences in body weight, blood glucose or insulin levels under a fasted state between the TG and age‐matched wild‐type mice (Figure S5C‐S5E). As anticipated, we observed a significant increase in miR‐29s in pancreatic islets (Figure 4b). TG mice were fed a chow diet because overexpression of miR‐29s in islets recapitulated the effect of the FFA‐mediated upregulation of miR‐29s expression. GSIS was not affected in this mouse model (Figure 4c). Indeed, miR‐29s have been reported to directly regulate insulin secretion by several studies, but a conclusive outcome has still not been reached (Dooley et al., 2016; Duan et al., 2019). miR‐29a was found to positively regulate insulin secretion in vivo, but an in vitro study revealed the opposite effect. We reasoned that this contradiction is likely due to the discrepant extent to which target genes were regulated under different experimental contexts. Monocarboxylate transporter‐1 (Mct1) and syntaxin‐1a (Stx1a), which are targeted by miR‐29a, are positive and negative regulators of insulin secretion, respectively. In our miR‐29s TG mice, a 2‐fold increase in miR‐29s could not markedly alter the expression of Mct1 or Stx1a (Figure S5F and S5G), which might account for the finding that insulin secretion secreted by high glucose was unaltered (Figure 4c). As anticipated, the levels of circulating miR‐29s were elevated in the plasma of miR‐29s TG mice (Figure 4d), which was associated with an increase in miR‐29s in the exosomal fraction (Figure 4e). As the intravenous administration of exosomal miR‐29s resulted in insulin resistance in mice, we assessed the insulin sensitivity of the miR‐29s TG mice by performing the i.p. GTT and ITT. Figure 4f shows that the TG mice were more hyperglycaemic at 15, 30, and 60 min. Consistently, the miR‐29 TG mice were more insulin resistant than their wild‐type littermates (Figure 4g). The role of miR‐29s in promoting insulin resistance was assayed by hyperinsulinaemic‐euglycaemic clamping of miR‐29s TG mice. The glucose infusion rate was significantly decreased in miR‐29s TG mice compared to wild‐type mice (Figure 4h), accompanied by a strong increase in hepatic glucose production in the liver (Figure 4i,j). This phenotype suggests that miR‐29s entered the liver and impaired insulin sensitivity. Thus, we tested the expression levels of miR‐29s in the liver and found them to be increased by 2‐fold in the livers of TG mice (Figure 4k), but hepatic endogenous transcription was not altered (Figure 4l). The levels of miR‐29s in skeletal muscle and adipose tissue were only slightly increased (Figure 4m,n). In HFD‐fed mice in which pancreatic miR‐29s were increased due to HFD feeding, we observed a similar pattern of miR‐29s expression in these three tissue types (Figure S5H‐S5J). Collectively, these results suggest that circulating miR‐29s from pancreatic β cells are preferentially taken up by the liver and decrease hepatic insulin sensitivity.

Next, we used a 3′‐biotin pull‐down assay of the reported target genes of miR‐29s in the insulin signalling pathway (akt, irs, pik3r1 and pik3r2 (Massart et al., 2017)) (Figure S5K) to evaluate the mechanistic basis of exosomal miR‐29s‐induced insulin resistance. miR‐29a bound the mRNA of akt, irs and pik3r1 but not that of pik3r3 (Figure S5L and S5M). Pik3r1 encodes p85α, which is the regulatory subunit of class IA phosphoinositide 3‐kinase (PI3K) and a validated target of miR‐29 family members (Park et al., 2009). Its expression level was reported to regulate the insulin signalling pathway (Yu et al., 1998). We confirmed that the knockdown of p85α expression in the liver promoted systemic insulin resistance (Figure S5N and S5O), indicating the importance of its regulatory effect on insulin action. Therefore, we selected p85α as the primary target for further study.

The protein level of p85α was significantly decreased in the livers of miR‐29s TG mice compared with wild‐type mice (Figures 4o and S6A). Consistently, insulin‐stimulated AKT activation was attenuated in the liver (Figures 4p and S6B), but that in skeletal muscle and adipose tissue was almost not affected (Figure S6C and S6D). Additionally, no overt enhanced lipogenesis or lipid accumulation was observed in miR‐29s TG mice, excluding the possibility that the impaired hepatic insulin resistance was due to lipid accumulation (Figure S6E and S6F). These results indicate that pancreatic β cell‐secreted miR‐29s are primarily delivered to the liver, where they impact insulin sensitivity and that the sustained delivery of miR‐29s to the liver may be an alternative mechanism underlying the etiopathogenesis of insulin resistance.

### 3.5 Pancreatic β cell‐derived miR‐29s targeted the liver and modulated hepatic insulin sensitivity

**FIGURE 5:**
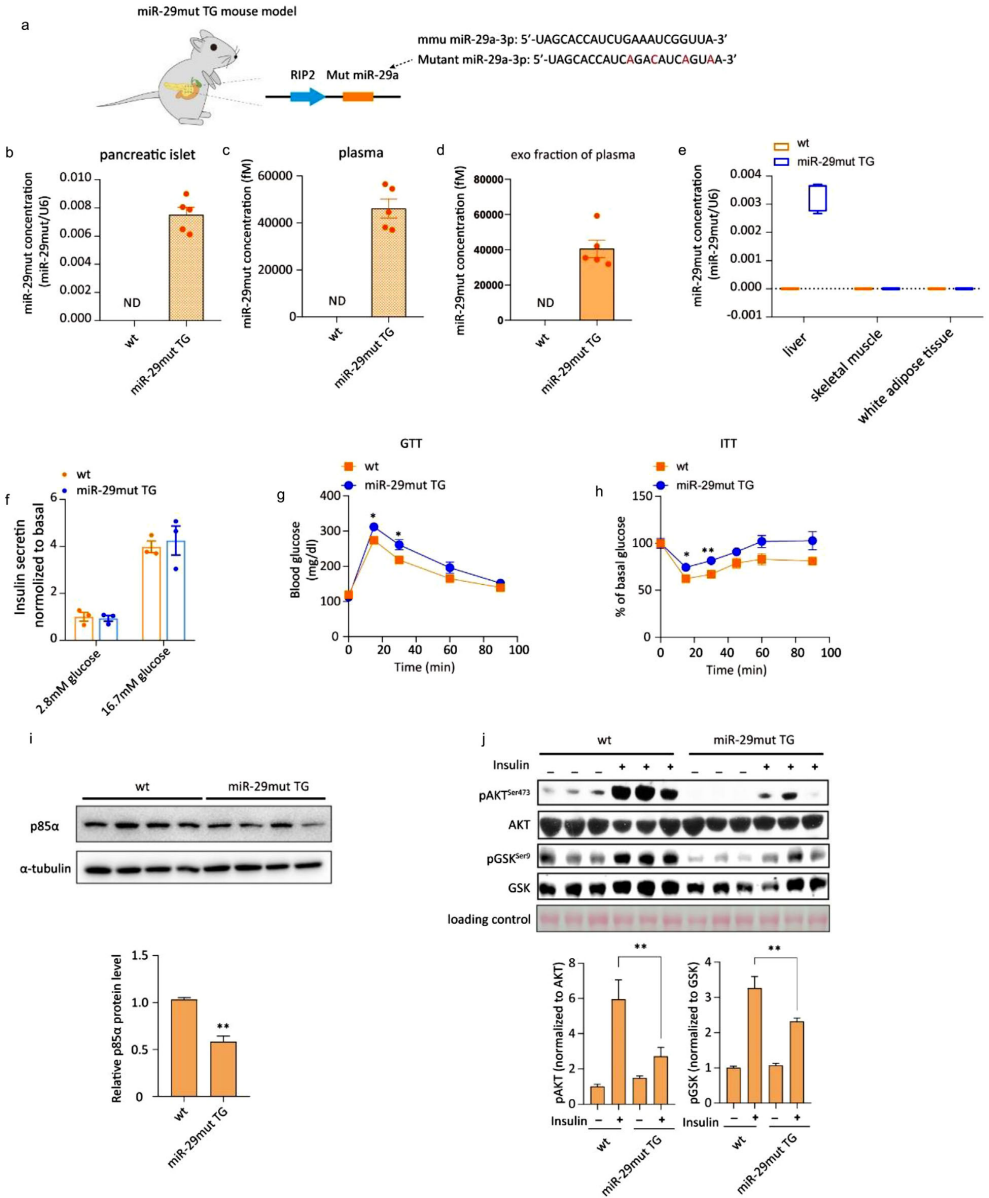
(a) Schematic of the generation of transgenic mice whose pancreatic β cells expressed mutant miR‐29a. (b‐d) qPCR analysis of the mutant miR‐29a concentrations in pancreatic islets (b), plasma (c) and exosomal fraction from the plasma of wild‐type or miR‐29mut TG mice. Islets from three mice were pooled together and represent a single spot (*n* = 15 for each group of mice whose pancreatic islets were analysed, *n* = 5 for each group of mice from whom plasma was analysed). Plasma was obtained from 15 mice in each group and pooled to harvest exosomes. qPCR analysis of mutant miR‐29a concentrations in the livers, skeletal muscle and gonadal adipose tissue of wild‐type or miR‐29mut TG mice (*n* = 5 for each group). (f) Glucose‐stimulated insulin secretion (GSIS) of cultured pancreatic islets from wild‐type or miR‐29mut TG mice. (g) The intraperitoneal glucose tolerance test (i.p. GTT) was administered to wild‐type or miR‐29mut TG mice (*n* = 15 for each group). (h) The insulin tolerance test (ITT) was administered to wild‐type or miR‐29mut TG mice (*n* = 8 for all groups). (i) Representative western blot of p85α and α‐tubulin in the livers of wild‐type or miR‐29mut TG mice (left panel). Analysis of p85α western blots from *n* = 3 independent experiments (right panel). (j) Representative western blot of insulin‐stimulated AKT phosphorylation in the livers of wild‐type or miR‐29mut TG mice (left panel). Analysis of western blots from *n* = 3 independent experiments (right panel)

To confirm whether pancreatic β cell‐derived miR‐29s target the liver, we tracked a foreign traceable miRNA expressed in β cells in vivo. This traceable miRNA was synthesized based on the miR‐29a sequence, with four nucleotides in the non‐seed region mutated (Figure 5a, highlighted in red). This mutant miR‐29a was easily distinguishable from native miR‐29s using a custom PCR probe (Figure S7A). Then, we designed a plasmid for the expression of mutant miR‐29a and selectively overexpressed this plasmid in the pancreatic β cells of mice (miR‐29mut TG mice) (Figure 5a). Pancreatic islet‐specific promoter leakiness was excluded (Figure S7B). The miR‐29mut TG mice also showed no difference in body weight, insulin secretion or glucose levels (Figure S7C‐S7E). To quantify the mutant miR‐29 in vivo, we serially diluted synthetic mutant miR‐29a and analysed it using qRT‐PCR assays to generate a standard curve. Concentrations of mutant miR‐29a in the pancreatic islets, plasma, liver, skeletal muscle and adipose tissue were calculated based on standard curves for each tissue type (Figure S7F‐S7H). As anticipated, a considerable level of mutant miR‐29a was detected in the pancreatic islets of miR‐29mut TG mice (Figure 5b). Mutant miR‐29a levels were also clearly detected in plasma, especially in exosomes isolated from the plasma of miR‐29mut TG mice (Figure 5c,d). Mutant miR‐29a was significantly increased in the liver compared with the skeletal muscle and adipose tissue of miR‐29mut TG mice, suggesting that secreted miR‐29s were preferentially delivered to the liver over these other organs (Figure 5e). GSIS in the mutant miR‐29a mouse model was not influenced by miRNA overexpression in the islets (Figure 5f).

Because the seed region of the sequence for the artificial mutant miR‐29a was intact, it could still theoretically target p85α mRNA and exert the same function as endogenous miR‐29a (Figure S7I). Therefore, we next investigated whether hepatic insulin sensitivity was affected by the secreted mutant miR‐29a. First, insulin sensitivity, as inferred from the GTT and ITT, was found to be impaired in miR‐29mut TG mice (Figure 5g,h). Protein levels of p85α were significantly decreased in the liver, indicating that the mutant miR‐29a was not only traceable but also functional (Figures 5i and S7J). PI3K forms a heterodimer composed of the p110 catalytic subunit and p85 regulatory subunit. The p85 regulatory subunit binds and stabilises the p110 catalytic subunit (Yu et al., 1998). Thus, we measured the protein levels of p110 to test whether a reduction in p85 would affect the activity of PI3K. As anticipated, decreased p85α levels led to the inhibition of p110α, further indicating that miR‐29s reduced the activity of PI3K (Figure S7K). Consistent with the result showing suppressed p85α expression, the insulin‐evoked phosphorylation of AKT and GSK was impaired in the miR‐29mut TG mice (Figures 5j and S7L). The mutant mice did not show enhanced lipid accumulation in the liver, ruling out defective lipid metabolism as the major cause of insulin resistance in miR‐29mut TG mice (Figure S7M). Collectively, by tracking the artificial mutant miR‐29a in miR‐29mut TG mice, we showed that pancreatic β cell‐derived miR‐29s predominantly target the liver. Notably, our data demonstrated that pancreatic β cell‐derived mutant miR‐29a inhibits hepatic insulin signalling, promoting insulin resistance.

### 3.6 Disruption of miR‐29s in pancreatic β cells improved hepatic insulin sensitivity in HFD‐fed mice

**FIGURE 6:**
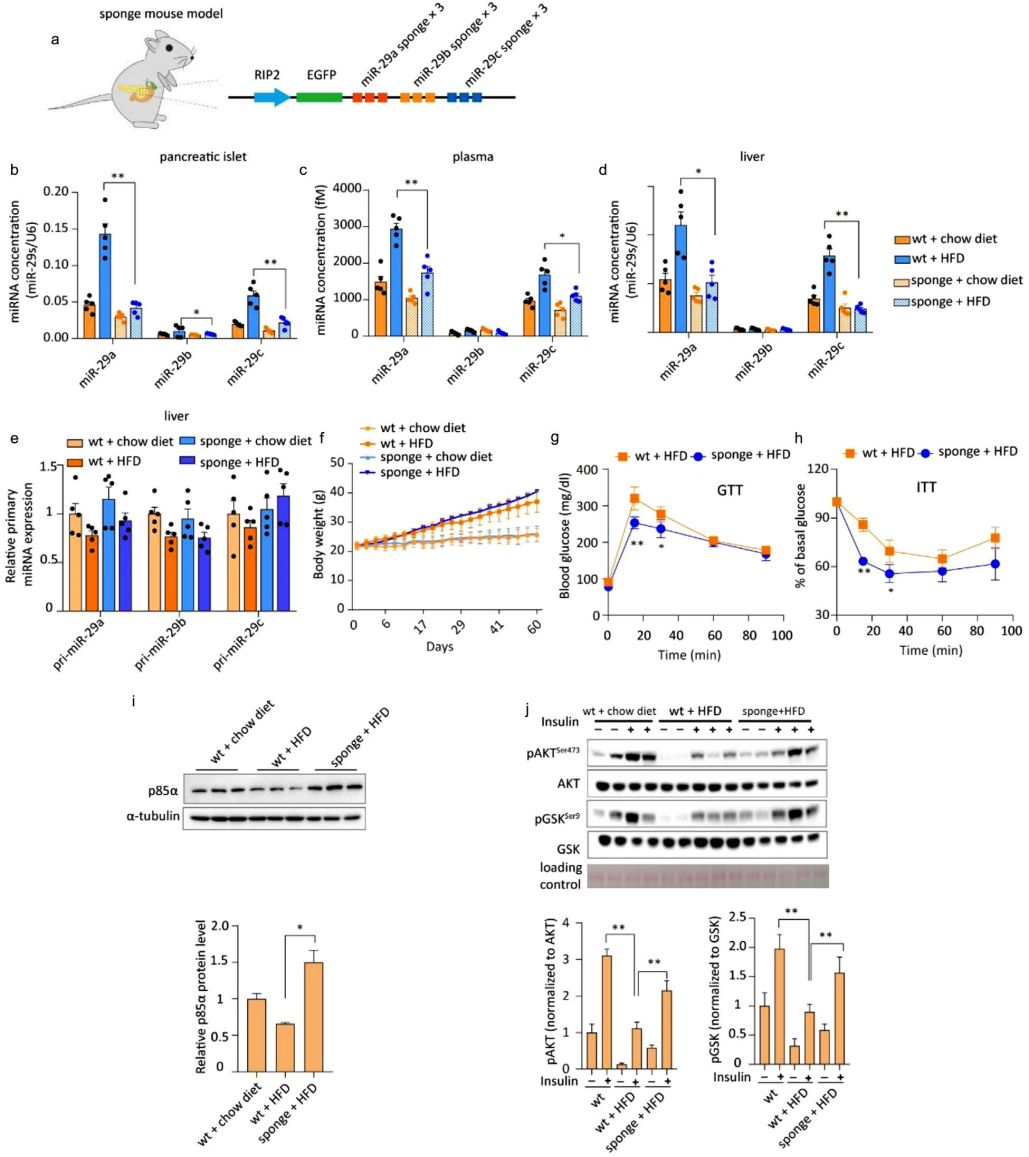
(a) Schematic of the design of transgenic mice whose pancreatic β cells overexpressed the miR‐29s sponge sequence. (b‐d) qPCR analysis of miR‐29s concentrations in the pancreatic islets (b), plasma (c), and livers (d) of the indicated groups. Islets from three mice were pooled together and represent a single spot (*n* = 15 for each group of mice whose pancreatic islets were analysed; *n* = 5 for each group of mice whose plasma was analysed). (e) qPCR analysis of primary miR‐29s levels in the livers of the indicated groups (*n* = 5 for each group). (f) Body weights of wild‐type mice and sponge mice fed a chow diet or a HFD (*n* = 15 for all groups). (g) The intraperitoneal glucose tolerance test (i.p. GTT) was administered to wild‐type and sponge mice (*n* = 15 for each group). (h) The insulin tolerance test (ITT) was administered to wild‐type or sponge mice fed a HFD (*n* = 8 for each group). (i) Representative western blot of p85α and α‐tubulin in the livers of wild‐type or sponge mice fed a HFD (left panel). Analysis of p85α western blots from *n* = 3 independent experiments (right panel). (j) Representative western blot of the insulin signalling pathway in the livers of wild‐type or sponge mice fed a HFD (left panel). Analysis of western blots from *n* = 3 independent experiments (right panel)

To investigate whether pancreatic β cell‐secreted miR‐29s contribute to insulin resistance in a pathological model, we next disrupted the secretion of miR‐29s in HFD‐fed mice. First, we generated a third type of TG mouse model—miR‐29s sponge (sponge) mice. This animal model expressed a sponge target construct designed following a previously successful strategy to compete with endogenous miR‐29s targets in pancreatic islets (Ma et al., 2011) (Figure 6a). Leakiness was excluded by identification of the tissue‐specific expression of the GFP DNA sequence (Figure S8A). Confocal images showed that the insertion cassette was successfully expressed in isolated pancreatic islets (Figure S8B). The physiological parameters of the sponge mice (insulin secretion, blood glucose levels and lipid accumulation) were not influenced by insertion of the miR‐29s sponge cassette (Figure S8C‐S8E). Then, the sponge mice were fed a HFD for 12 weeks at weaning. HFD feeding resulted in an approximately 3‐fold increase in miR‐29s in the pancreatic islets of control mice (Figure 6b). However, the HFD‐stimulated upregulation of miR‐29s was almost completely abrogated in the sponge mice (Figure 6b). A significant reduction in miR‐29s was found in the plasma and livers of sponge mice compared with HFD‐fed control mice (Figure 6c,d). Importantly, endogenous levels of miR‐29s transcription were not changed in the livers of either control or sponge mice on a HFD, confirming that the increased miR‐29s levels in the livers of HFD‐fed control mice originated within pancreatic β cells (Figure 6e). Body weight gains were similar between the sponge mice and control group (Figure 6f). Typically, a HFD leads to hyperglycaemia and insulin resistance in mice. However, compared to wild‐type mice, sponge mice exhibited more rapid glucose clearance and improved insulin sensitivity on a HFD (Figure 6g,h). Compared with wild‐type controls on a chow diet, sponge mice fed a chow diet showed no difference in glucose homoeostasis (Figure S8F and S8G). As expected, the HFD‐mediated reduction in p85α expression levels in the liver was reversed in sponge mice (Figures 6i and S8H), associated with improved insulin signalling (Figures 6j and S8I). Altogether, these results demonstrate that pancreatic β cell‐derived miR‐29s contribute to HFD‐induced insulin resistance.

We also confirmed the origin of the increased circulating miR‐29s in mice under fasting conditions using sponge mice. Compared to wild‐type mice, which exhibited increased circulating miR‐29a (Figure 2k), fasted sponge mice did not exhibit increased circulating miR‐29s, indicating that the increase in circulating miR‐29s likely originated in pancreatic β cells (Figure S8J).

### 3.7 Secreted miR‐29s from FFA‐treated pancreatic islets blunted in vitro hepatic insulin sensitivity and altered glucose output

**FIGURE 7:**
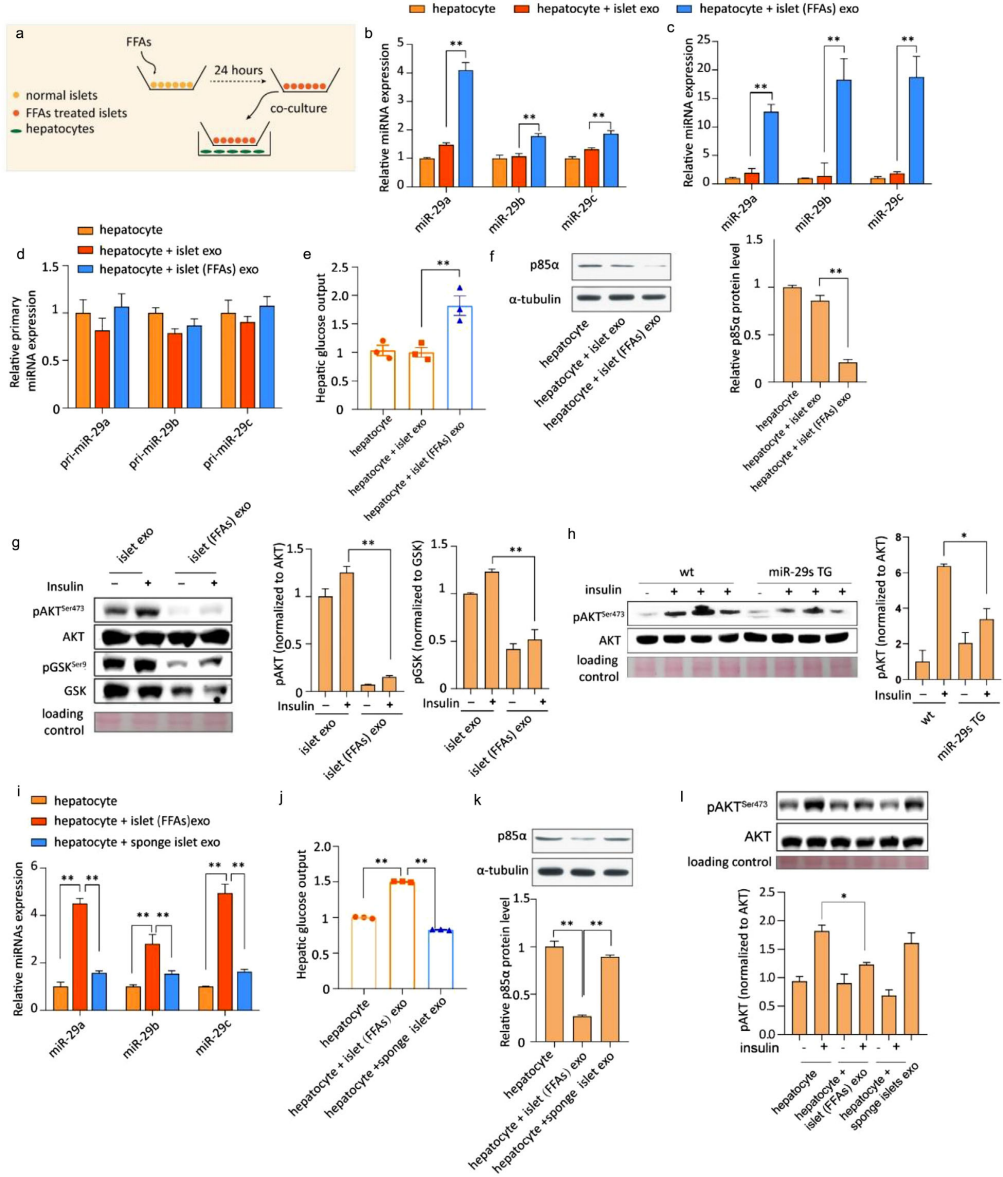
(a) Schematic of the co‐culture system. (b) qPCR analysis of the expression of miR‐29s in untreated hepatocytes or hepatocytes co‐cultured with either normal islets or FFA‐treated islets. Data are from *n* = 3 independent experiments. (c) qPCR analysis of the expression of miR‐29s in untreated hepatocytes or hepatocytes treated with exosomes from either normal islets or FFA‐treated islets. Data are from *n* = 3 independent experiments. (d) qPCR analysis of the expression of primary miR‐29s in the groups indicated in panel C. (e) Glucose output assay of hepatocytes treated as indicated in panel C. (f) Representative western blot of p85α and α‐tubulin in hepatocytes treated as indicated in panel C (left panel). Analysis of p85α western blots from *n* = 3 independent experiments (right panel). (g) Representative western blot of the insulin signalling pathway in hepatocytes treated as indicated in panel C (left panel). Analysis of western blots from *n* = 3 independent experiments (right panel). (h) Representative western blot of insulin‐stimulated AKT phosphorylation in hepatocytes isolated from wild‐type or miR‐29s TG mice (left panel). Analysis of western blots from *n* = 3 independent experiments (right panel). (i) qPCR analysis of the expression of miR‐29s in untreated hepatocytes or hepatocytes treated with exosomes from either FFA‐treated islets or sponge islets. Data are from *n* = 3 independent experiments. (j) Glucose output assay of hepatocytes treated as indicated in panel I. (k) Representative western blot of p85α and α‐tubulin in hepatocytes treated as indicated in panel I (upper panel). Analysis of p85α western blots from *n* = 3 independent experiments (bottom panel). (l) Representative western blot of insulin‐stimulated AKT phosphorylation in hepatocytes treated as indicated in panel I (upper panel). Analysis of p85α western blots from *n* = 3 independent experiments (bottom panel)

**FIGURE 8:**
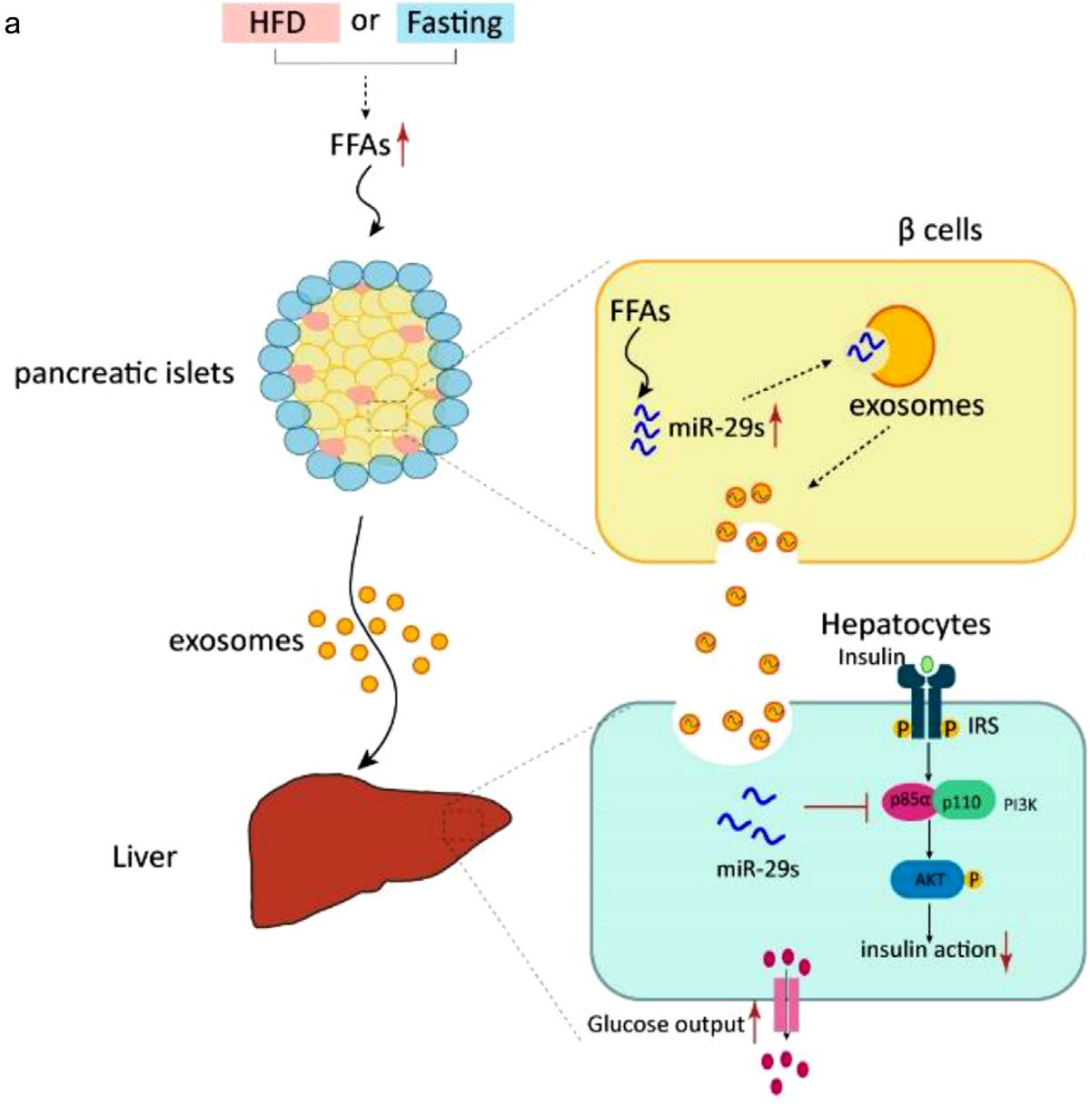
(a) Schematic showing the mechanism underlying the attenuation of insulin sensitivity in the liver by pancreatic islet‐derived miR‐29s

We investigated the transferability and functionality of exosomal miR‐29s in vitro with a co‐culture system in which hepatocytes were incubated with pancreatic islets with or without FFA pre‐treatment (Figure 7a). We observed increased miR‐29s levels in hepatocytes co‐cultured with FFA‐treated islets. However, in hepatocytes co‐cultured with non‐stimulated islets, a moderate but not significant increase in miR‐29s was detected, confirming our hypothesis that the miR‐29‐mediated pathway was selectively activated by FFAs (Figure 7b). Additionally, by treating hepatocytes with exosomes derived from islets with or without FFA stimulation, we demonstrated that the secreted miR‐29s were predominantly transferred to the liver by exosomes (Figure 7c,d). Fluorescently labelled miRNAs were loaded into exosomes to illustrate the delivery of miRNAs via exosomes incubated with hepatocytes. Using confocal microscopy analysis, we confirmed that labelled miRNAs were delivered into hepatocytes (Figure S9A). Moreover, the treatment of hepatocytes with FFA‐induced islet exosomes enhanced their glucose output (Figure 7e). Next, we measured the protein level of p85α to assess the biological function of exogenous miR‐29s delivered by islet exosomes. Incubation with FFA‐treated islet exosomes reduced the expression level of p85α in hepatocytes. Conversely, incubation with exosomes from non‐stimulated islets did not affect p85α expression (Figure 7f). Consistently, insulin‐stimulated AKT activation was affected by FFA‐treated islet exosomes (Figure 7g). Notably, hepatocytes isolated from miR‐29s TG mice also showed impaired insulin‐stimulated AKT activation (Figure 7h). To further confirm that pancreatic β cell‐released miR‐29s promoted FFA‐induced hepatic insulin resistance, we collected exosomes from FFA‐treated primary cultured islet cells of wild‐type and sponge mice. As shown in Figure 7i, when hepatocytes were incubated with sponge islet exosomes, miR‐29s levels were not upregulated, in contrast to hepatocytes treated with FFA‐induced exosomes. The increased p85α level rescued the enhanced hepatic glucose output induced by FFAs and insulin‐stimulated AKT activation in hepatocytes treated with sponge mouse‐derived exosomes (Figure 7j‐l). These results directly demonstrate that a high level of FFAs induced pancreatic islet miR‐29s secretion via exosomes, thereby contributing to the downregulation of hepatic insulin sensitivity. As shown in Figure 8, our study reveals that high levels of FFAs, triggered by either pathological (a HFD) or physiological (fasting) conditions, stimulate pancreatic β cells to secrete miR‐29s that enter the circulation via exosomes. These exosomes are predominantly delivered to the liver, where they limit insulin sensitivity.

## 4 DISCUSSION

Organ crosstalk has been shown to occur via the transmission of exosomal miRNAs. Almost all cell types can secrete exosomes, but the range of their exosomal miRNA cargo varies under different conditions (Zhang et al., 2010). Thus, exosomal miRNAs can be used as not only diagnostic markers of disease but also molecular mediators of physiological and pathological processes.

The pancreas is an essential secretory organ. However, the secretion of miRNAs from the pancreas was not investigated until very recently. A study reported that β cell‐secreted miR‐26s influenced peripheral insulin sensitivity (Xu et al., 2020). Herein, we found that pancreatic β cells respond to high levels of FFAs by secreting exosomal miR‐29s to target the hepatic insulin signalling pathway and promote insulin resistance. Thus, our study confirmed that pancreatic islets release not only conventional hormones but also miRNAs via the secretion of exosomes.

Additionally, we identified a new factor—miR‐29s—to be an essential regulator of glucose homeostasis. The local production of miR‐29s has been proposed to regulate insulin sensitivity. Here, we provide three lines of evidence that exosomal miR‐29s promote insulin resistance in the liver. First, exosomal miR‐29s originating from β cells caused systemic insulin resistance in miR‐29s TG mice (Figure 4), miR‐29mut mice (Figure 5) and mice intravenously injected with exosomal miR‐29s (Figure 3). Furthermore, preventing the secretion of miR‐29s from β cells (sponge mice) rescued glucose intolerance in HFD‐fed mice (Figure 6). Second, we revealed that the role of exosomal miR‐29s in systemic insulin resistance depends on its effects on the liver. We used three complementary mouse models to demonstrate that islet‐derived exosomal miR‐29s are primarily and preferentially delivered to the liver. Consistent with this hepatic selectivity, insulin‐stimulated AKT activation was significantly impaired in only the liver. Finally, in vitro experiments further confirmed that islet‐derived exosomal miR‐29s entered hepatocytes and impaired insulin action. These findings have uncovered a novel islet‐derived factor that actively contributes to glucose homeostasis.

***miR‐29s were selectively sorted into exosomes in response to high levels of FFAs*** . Our results show the uniqueness of pancreatic islet responses to high levels of FFAs, as pancreatic islets selectively released miR‐29s over other miRNAs, and that this effect is markedly potentiated by either obesity or fasting (Jiang & Zhang, 2003). Previous studies have revealed that miRNAs can be differently sorted into exosomes in the context of specific physiological or pathological states. This is conceptually similar to the secretion of conventional hormones, which exhibit stimulus‐secretion coupling, such as GSIS (Roder et al., 2016).

The selectivity of exosomal miR‐29s regulation and secretion by islets is indicative that the pattern of cell types that respond to given stimuli is context‐dependent. FFAs are natural ligands essential to vesicle exocytosis, such as GSIS. FFAs bind their receptor FFAR1/GPR40, which is highly expressed in β cells, and cause an increase in intracellular Ca^2+^ levels, promoting exocytosis (Fujiwara et al., 2005; Nolan et al., 2006). This evidence prompted us to posit that the secretion of miRNAs from islets may exhibited some mechanistic similarities with the process of insulin secretion and that miRNA secretion from islets is enabled by FFAs in β cells. We also speculate that as the secretion induced by FFAs reduced the levels of miR‐29s in β cells, such secretion may trigger an increase in the transcription of local miR‐29s. An alternative or parallel explanation is that FFAs can directly increase the transcription of miR‐29s in pancreatic islets. FFAs triggered a sharp reduction in c‐Myc (Ayala‐Torres et al., 1997; Thompson, 1998), which can bind both miR‐29b‐1/a and miR‐29b‐2/c in the vicinity of the transcription start site and negatively regulate miR‐29 expression (Kriegel et al., 2012).

***Defining target tissues of β cell‐derived exosomal miR‐29s*** . In our study, we observed an interesting pattern of β cell‐derived miR‐29s distribution. We confirmed that miR‐29s were primarily taken up by the liver, as shown by tracking mutant miR‐29s, which were distinguishable from endogenous miR‐29s. Tracking miRNAs in vivo is not without its challenges and is made difficult by the lack of feasible methods. Several approaches have been developed to track exosomes in vivo, including the injection of labelled exosomes or the Cre‐loxp‐mediated colour switching of recipient cells, have been developed (Zomer et al., 2015). However, these methods fail to follow exosomes between organs in vivo. Additionally, the abolishment of miRNA genesis by Dicer knockout has also been utilized to track miRNAs (Thomou et al., 2017). However, the global ablation of miRNAs is not suitable for a study focused on the functions of a specific miRNA. We observed considerable amounts of mutant miR‐29a in the liver. Specifically, 0.032 fmol of mutant miR‐29s per μg of total liver RNA, which is equivalent to 966 copies per cell, was observed. It has been reported that endogenous mammalian miRNAs are dynamically expressed at copy numbers ranging from 1 to 10,000 per cell.

Moreover, miRNAs expressed at levels below 100 copies per cell are not believed to be targets for repression given the low possibility of encountering a target‐containing transcript. Thus, pancreatic β cell‐derived miR‐29s are detectable in the liver at functional levels. However, in other insulin resistance‐associated organs, only a small amount of mutant miR‐29a was detected.

Defining the targets of exosomes remains another challenge in this field. Exosomal membrane markers differ from cell to cell; this feature may facilitate target cell selectivity (Horibe et al., 2018; Hoshino et al., 2015). In light of previous studies, we reasoned that islet exosomes might also bear tissue‐specific membrane markers that can mediate their preferential delivery to the liver. By promoting the differential internalisation of exosomes, these membrane markers may determine the final expression and/or function of exosomal miRNAs in recipient cells. Additionally, the mechanism of exosome uptake may determine the effectiveness of the entry of these exogenous miRNAs and their fates. Exosomes taken up by membrane fusion can release miRNAs/mRNAs into recipient cells, in which they promote specific cellular responses. Exosomes are taken up via micropinocytosis, phagocytosis or endocytosis and can release their miRNAs into recipient cells. Their exogenous genetic bio‐cargo may either undergo clearance or promote a cellular response (Gonda et al., 2019; McKelvey et al., 2015). Therefore, the final target and effect of exosomal miRNAs can be determined by two factors: a) membrane protein‐mediated selective uptake and/or b) the fate of exosomal miRNAs in different cell types. Horibe et al. also showed that the capacity of exosome uptake differed depending on the recipient cell but not the donor cell (Horibe et al., 2018). These facts may explain why we observed such a discrete distribution pattern of islet‐derived exosomal miR‐29s in vivo. We think more studies to address these profound questions will be necessary. Improved EV tracking technology may facilitate the direct visual observation of exosome transfer between organs in vivo and subsequently provide a better understanding of exosomal miRNA transfer and function.

***The mechanism underlying the effect of exosomal miR‐29s*** . A known target of miR‐29s is p85α, the regulatory subunit of PI3K. We observed that the inhibition of p85α caused a significant insulin resistance. A reduction in p85α could arguably lead to an unclear result, as it could conceivably improve or impair insulin sensitivity (Taniguchi et al., 2007; Ueki et al., 2003). P85α monomers are more abundant than p110, and inactive monomers compete with p85‐p110 heterodimers to bind IRS. As a possible explanation for this paradox, when monomeric p85α is preferentially reduced, the heterodimer might then be allowed to bind the phosphorylated IRS protein, maintaining normal insulin signalling. Conversely, insulin resistance might develop when p85 reduction leads to the inhibition of p110 (Taniguchi et al., 2006). In our study, we observed a reduction in p85 accompanied by a decrease in p110, which is consistent with previous research showing that miR‐29s inhibit PI3K activity (Dooley et al., 2016). miR‐29s have multiple targets in the insulin signalling pathway. Our results also confirmed that miR‐29s bind Irs1 and AKT. Additionally, our data showed that p85α plays a role in insulin sensitivity (Figure S5N and S5O).

Taken together, our results provide new insight into secretory factors from pancreatic β cells implicated in the regulation of glucose homeostasis. Additionally, we have discovered a new pancreatic endocrine factor that mediates crosstalk between the pancreas and liver. Chronic obesity‐associated high levels of FFAs are a significant pathological stimulus that contributes to insulin resistance (Shoelson et al., 2006). Finally, we provide an alternative mechanism underlying FFA‐induced insulin resistance.

## CONFLICT OF INTEREST STATEMENT

The authors have declared that no conflict of interest exists

## AUTHOR CONTRIBUTIONS

Jing Li, Yujing Zhang, Ke Zen, and Chen‐Yu Zhang conceived and designed the study. Jing Li, Yujing Zhang, Yangyang Ye, Dameng Li, Yuchen Liu, Eunyoung Lee, Mingliang Zhang, Xin Dai, Xiang Zhang, and Shibei Wang acquired data. Jing Li, Yujing Zhang, Xiaohong Jiang, Weiping Jia, and Antonio Vidal‐Puig analysed and interpreted data. Jing Li, Yujing Zhang, Antonio Vidal‐Puig, Xiaohong Jiang, and Chen‐Yu Zhang drafted the manuscript. Jing Li, Chen‐Yu Zhang, and Junfeng Zhang obtained funding. Chen‐Yu Zhang, Antonio Vidal‐Puig, and Ke Zen supervised the study. All authors reviewed and approved the manuscript.

## Supporting information

Supporting information.
