# Supplementary material for: Pancreatic β cells control glucose homeostasis via the secretion of exosomal miR‐29 family
Source: J Extracell Vesicles. 2021 Jan 21;10(3):e12055. doi: 10.1002/jev2.12055 (PMC7820156; doi:10.1002/jev2.12055)
Supplement: Supplementary file 1 — Supporting information. [file JEV2-10-e12055-s001.docx]

**Pancreatic β cells control glucose homeostasis via the secretion of exosomal miR-29 family**

Supplementary Figure 1


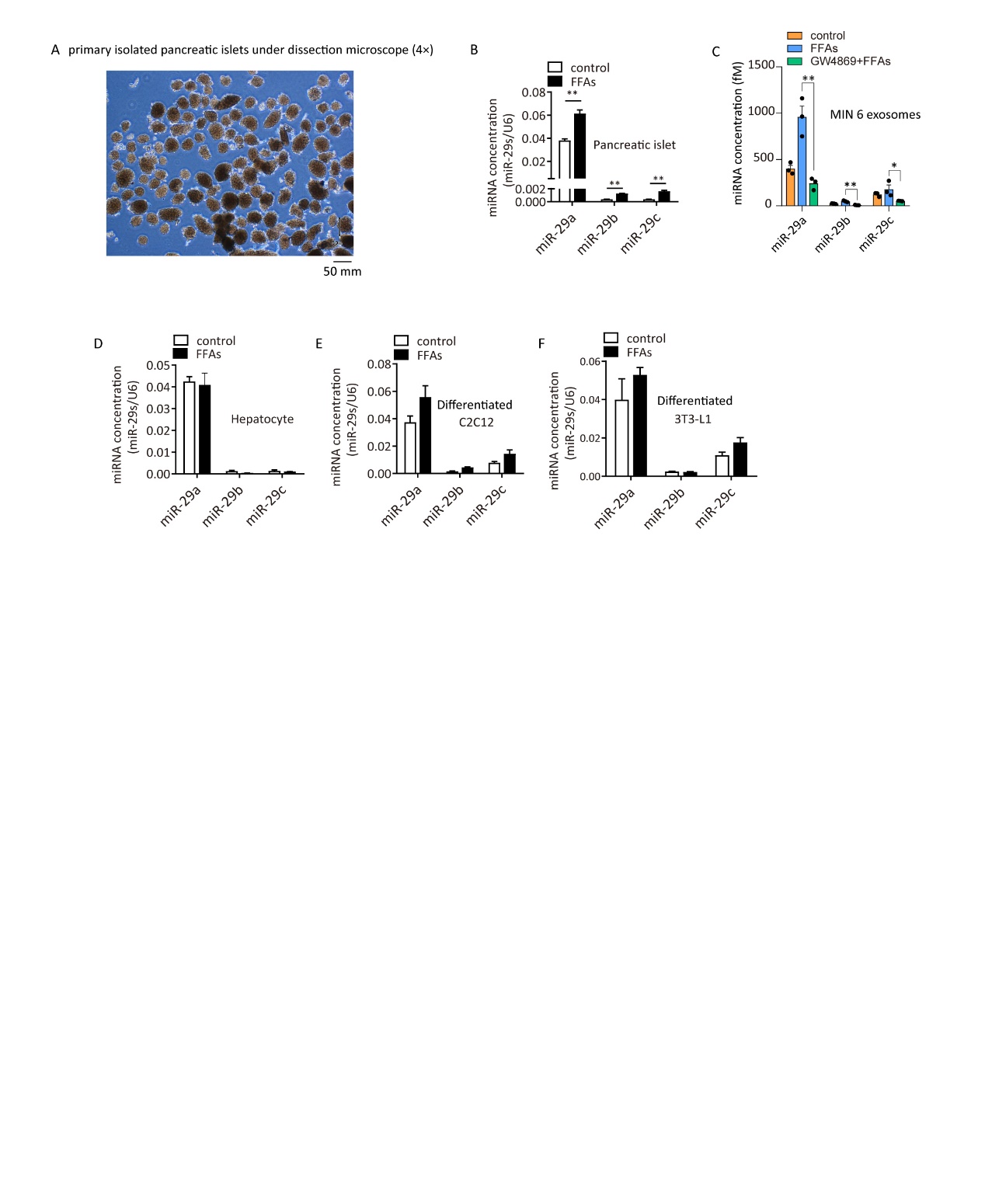


(A). Images of primary isolated pancreatic islets from mouse

(B). qPCR analysis for the miR-29s concentrations in untreated or FFAs-treated pancreatic islets.

(C). qPCR analysis for the miR-29s concentrations in untreated, FFAs-treated or FFAs-treated plus

GW4869.

(D). qPCR analysis for the miR-29s concentrations in untreated or FFAs-treated hepatocytes.

(E). qPCR analysis for the miR-29s concentrations in untreated or FFAs-treated dirrerentiated

C2C12 cells.

(F). qPCR analysis for the miR-29s concentrations in untreated or FFA-treated dirrerentiated

3T3-L1 cells.

Error bars indicate SE (*p < 0.05; **p < 0.01).

**Table S1. Demographic information for the obese diabetic patients**

| **Number** | **BMI** | **Gender** | **Age** |
| --- | --- | --- | --- |
| **1** | **33.35** | **Male** | **65** |
| **2** | **31.14** | **Male** | **58** |
| **­3** | **29.4** | **Male** | **58** |
| **4** | **28.34** | **Female** | **47** |
| **5** | **28.31** | **Male** | **62** |
| **6** | **28.28** | **Female** | **54** |
| **7** | **27.48** | **Female** | **20** |
| **8** | **27** | **Male** | **53** |
| **9** | **26.73** | **Male** | **51** |
| **10** | **26.67** | **Male** | **45** |
| **11** | **26.03** | **Female** | **71** |
| **12** | **26.03** | **Male** | **65** |
| **13** | **26** | **Male** | **69** |
| **Average** | **26.5±0.7** | **------** | **------** |

Supplementary Figure 2


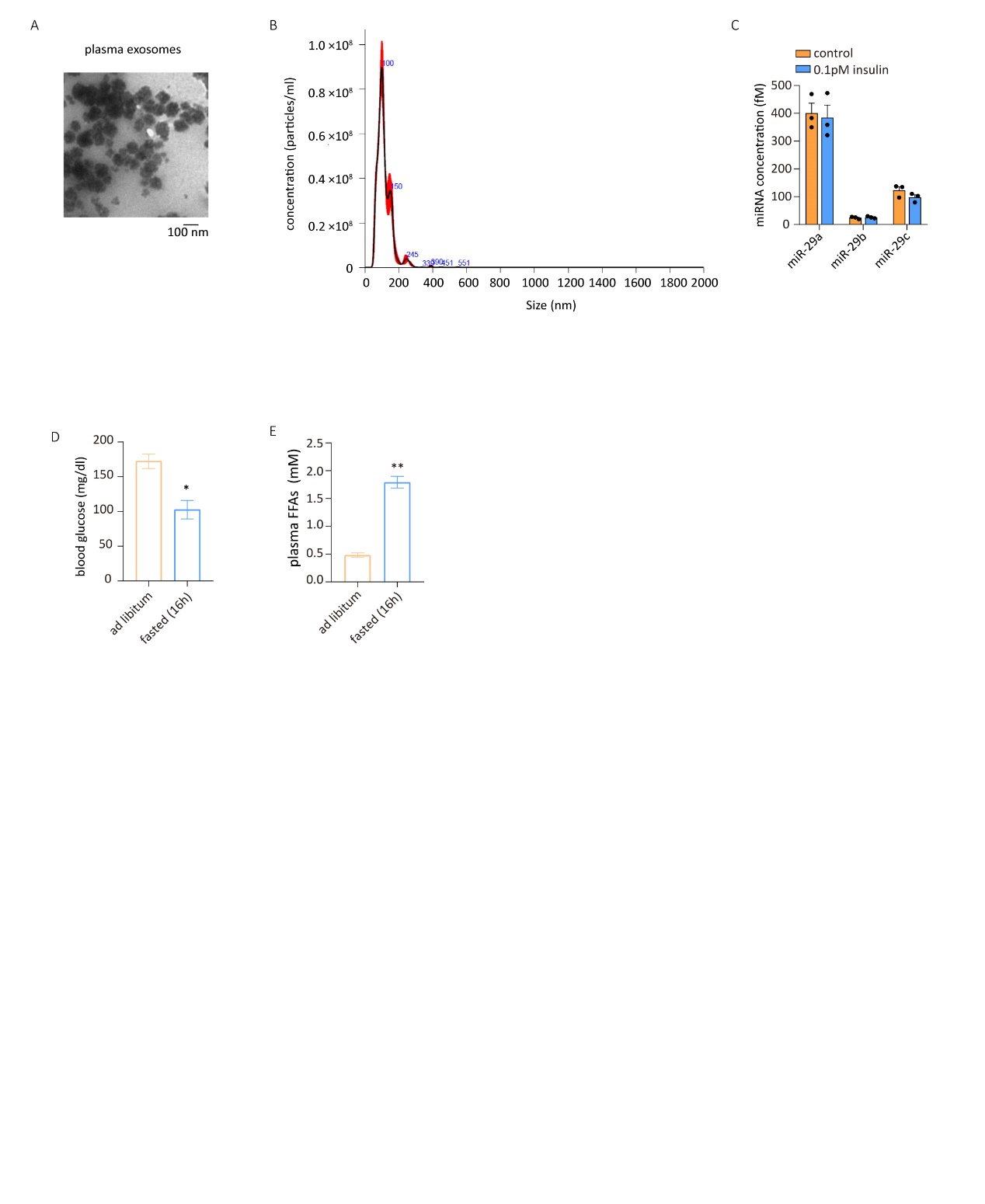


(A). The TEM image of exosomes isolated from the plasma of *ob/ob* mice.

(B). Nanoparticle tracking analysis (NTA) of exosomes isolated from the plasma of *ob/ob* mice.

(C). qPCR analysis for the miR-29s concentrations in exosomes isolated from MIN 6 cells untreated or treated with high levels of insulin for 24 h.

(D). Blood glucose levels of mice after fasted for 16 h (n = 5 for each group).

(E) The levels of FFAs in plasma of mice on ad libitum diet (n = 13) or fasted (n = 14) for 16 h.

Error bars indicate SE (*p < 0.05; **p < 0.01).

Supplementary Figure 3


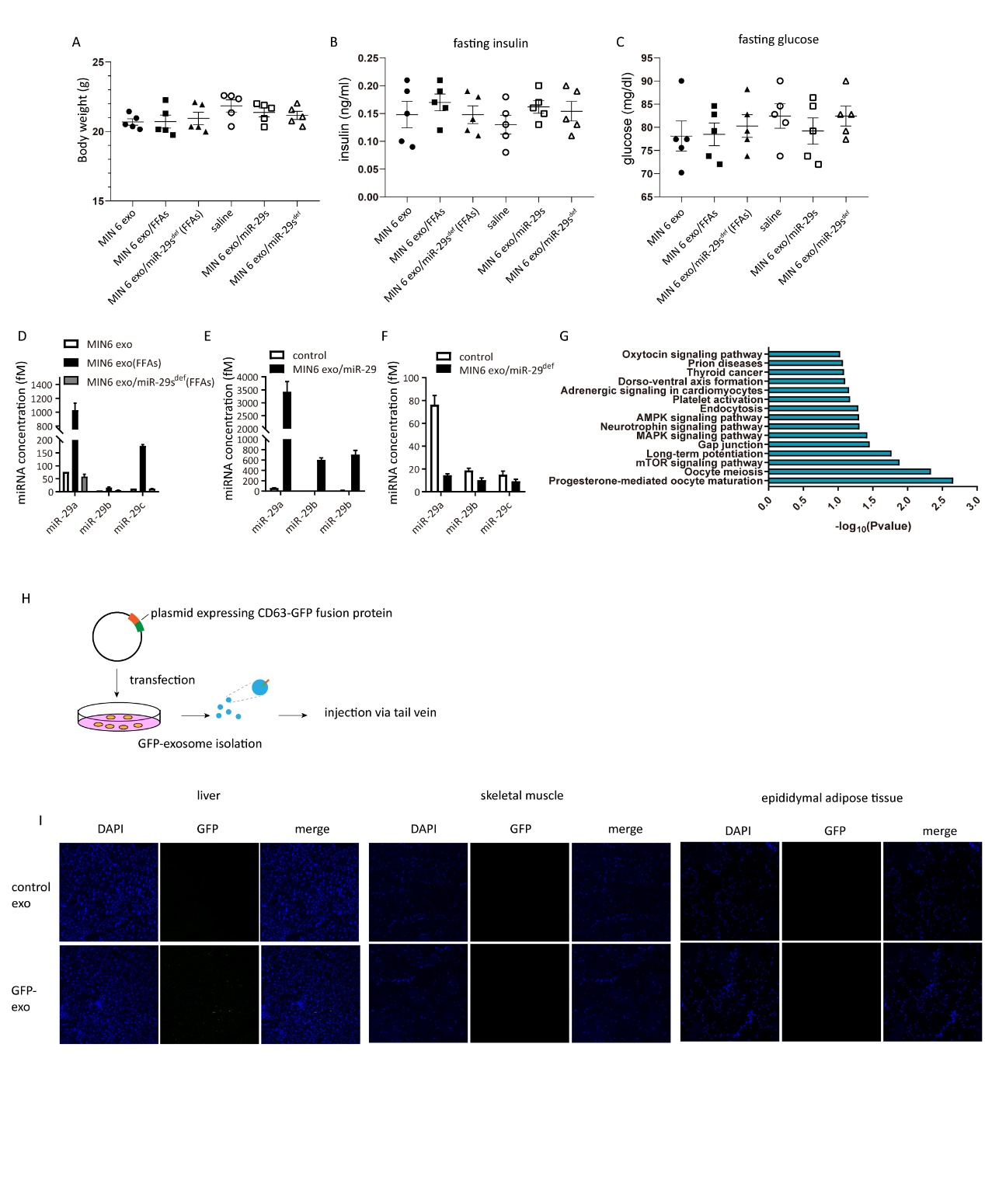


(A). Body weight of mice after exosome injection.

(B). Insulin of mice after exosome injection. Plasma insulin levels were measured after fasted 16 h.

(C). Blood glucose levels of mice after exosome injection. Plasma insulin levels were measured after fasted 16 h.

(D). qPCR analysis for the miR-29s concentrations in exosomes produced by MIN 6 cells untreated or treated with FFAs for 24 h; or produced by MIN 6 knocked down miR-29s and treated with FFAs for 24 h (FFA+antimiR-29s).

(E). qPCR analysis for the miR-29s concentrations in exosomes produced by MIN 6 cells transfected with scrambled mimetic ncRNA (control) or mimic miR-29s (MIN 6/miR-29).

(F). qPCR analysis for the miR-29s concentrations in exosomes produced by MIN 6 cells transfected with scrambled anti-ncRNA (control) or anti-miR-29s (MIN 6/miR-29^def^).

(G). KEGG pathway analysis of proteins of exosome from FFAs-treated MIN 6 cells.

(H). schematic of plasmid expressing CD63-GFP fusion protein and the produce of GFP-labelled exosomes.

(I). Confocal images of GFP expression in livers, skeletal muscles and gonadal skeletal muscles of mice after exosome injection.

Error bars indicate SE (*p < 0.05; **p < 0.01).

Supplementary Figure 4


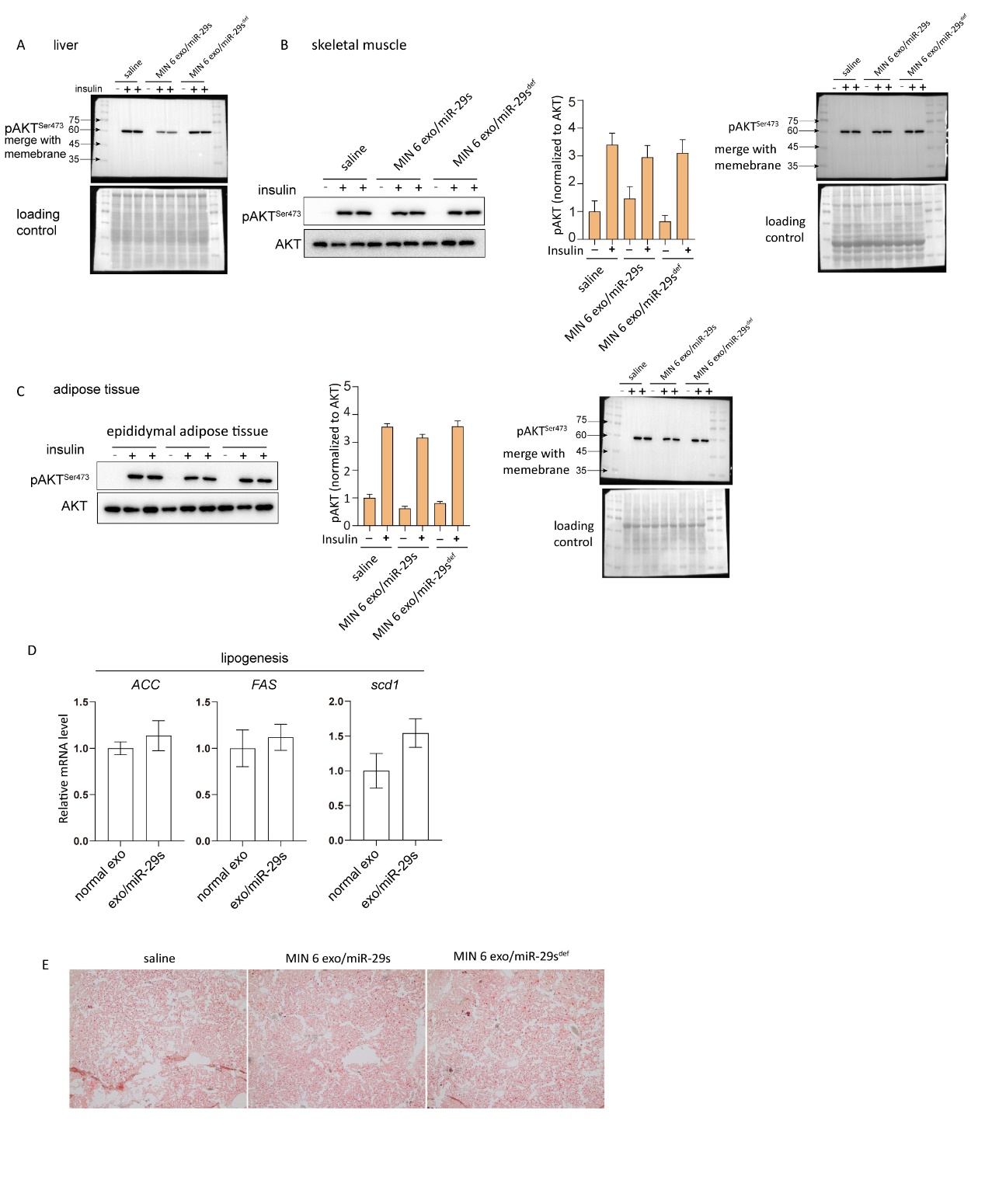


(A). The uncropped membrane of phosphorylated AKT and loading control for figure 3G. Ponceau staining of the uncropped membrane was used as loading control. The target western blot bands were obtained and merged with the image of uncropped membrane.

(B-C). Representative western blot of insulin-stimulated AKT phosphorylation in the skeletal muscles (B) and gonadal adipose tissues (C) of mice injected with saline, exosomes replete with or depleted with miR-29s (left panel). Analysis of western blots from n = 3 independent experiments (middle panel). Ponceau staining of the uncropped membrane was used as loading control. The target western blot bands were obtained and merged with the image of uncropped membrane (right panel).

(D). The mRNA levels of lipogenesis genes in the livers of mice administrated with normal exosomes or exosomes replete with miR-29s.

(E). Histochemistry analysis of lipid accumulation in livers of mice injected with saline, exosomes replete with or depleted with miR-29s. The liver sections were subjected to H&E staining and Oil-Red-O staining.

Supplementary Figure 5


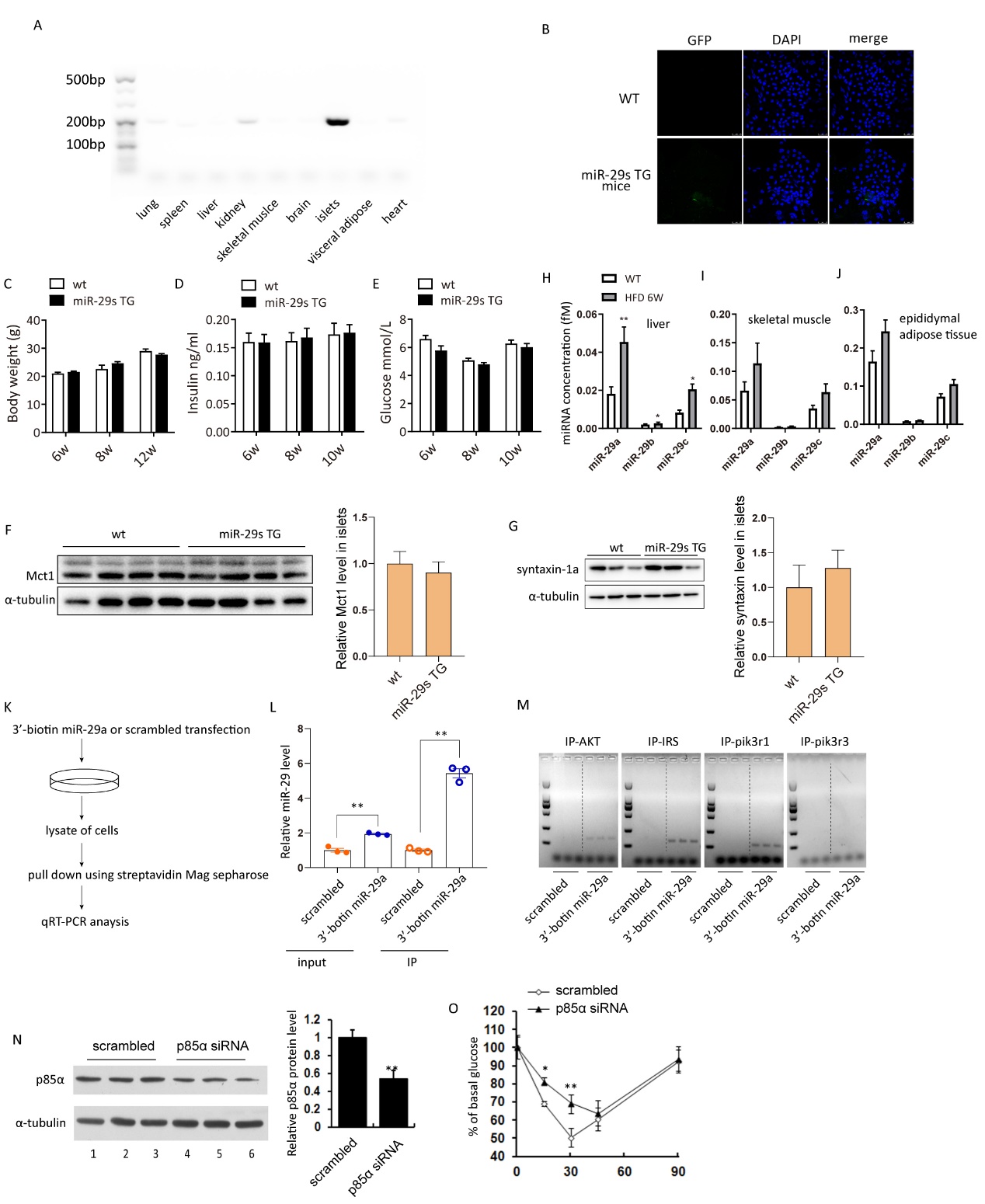


(A). Semi quantitative RT-PCR analysis of GFP expression in various tissues. GFP and miR-29a/b/c were both under the control of RIP2 promoter, GFP serveed as an indicator to test the tissue specific of insert miR-29a/b/c.

(B). Confocal microscopy images of pancreatic islets derived from wild-type or miR-29s TG mice.

(C). Body weight of wild-type (chow diet) or miR-29s TG mice (chow diet) at 6-week, 8-week, and 12-week.

(D). Plasma insulin levels of wild-type (chow diet) or miR-29s TG mice (chow diet) at 6-week, 8-week, and 12-week as determined by Elisa. Mice were fasted overnight and then subjected to blood extracting.

(E). Glucose levels of wild-type (chow diet) or miR-29s TG mice (chow diet) at 6-week, 8-week, and 12-week. Mice were fasted overnight before test the blood glucose.

(F-G). Representative western blot of insulin releasing regulator mct-1 (F) and syntaxin-1a (G) (left panel). Analysis of western blots from n = 3 independent experiments (right panel)

(H-J). qPCR analysis for the miR-29s concentrations in livers (H), skeletal muscles (I) and adipose tissues (J) (n=5 for each groups).

(K). The schematic of 3’-bitoin miRNA pull down assay.

(L). The level of miR-29a in cells transfected with 3’-biotin miR-29a (input) and in immunoprecipitants (IP).

(M). The levels of mRNAs in immunoprecipitants as determined by agarose gel electrophoresis following the pull-down assay in L.

(N). Representative western blot of p85 α in the livers of mice intravenously administrated with scrambled or p85 α siRNA (left panel). Analysis of western blots from n = 3 independent experiments (right panel).

(O). Insulin tolerance test of mice in N.

Error bars indicate SE (*p < 0.05; **p < 0.01).

Supplementary Figure 6


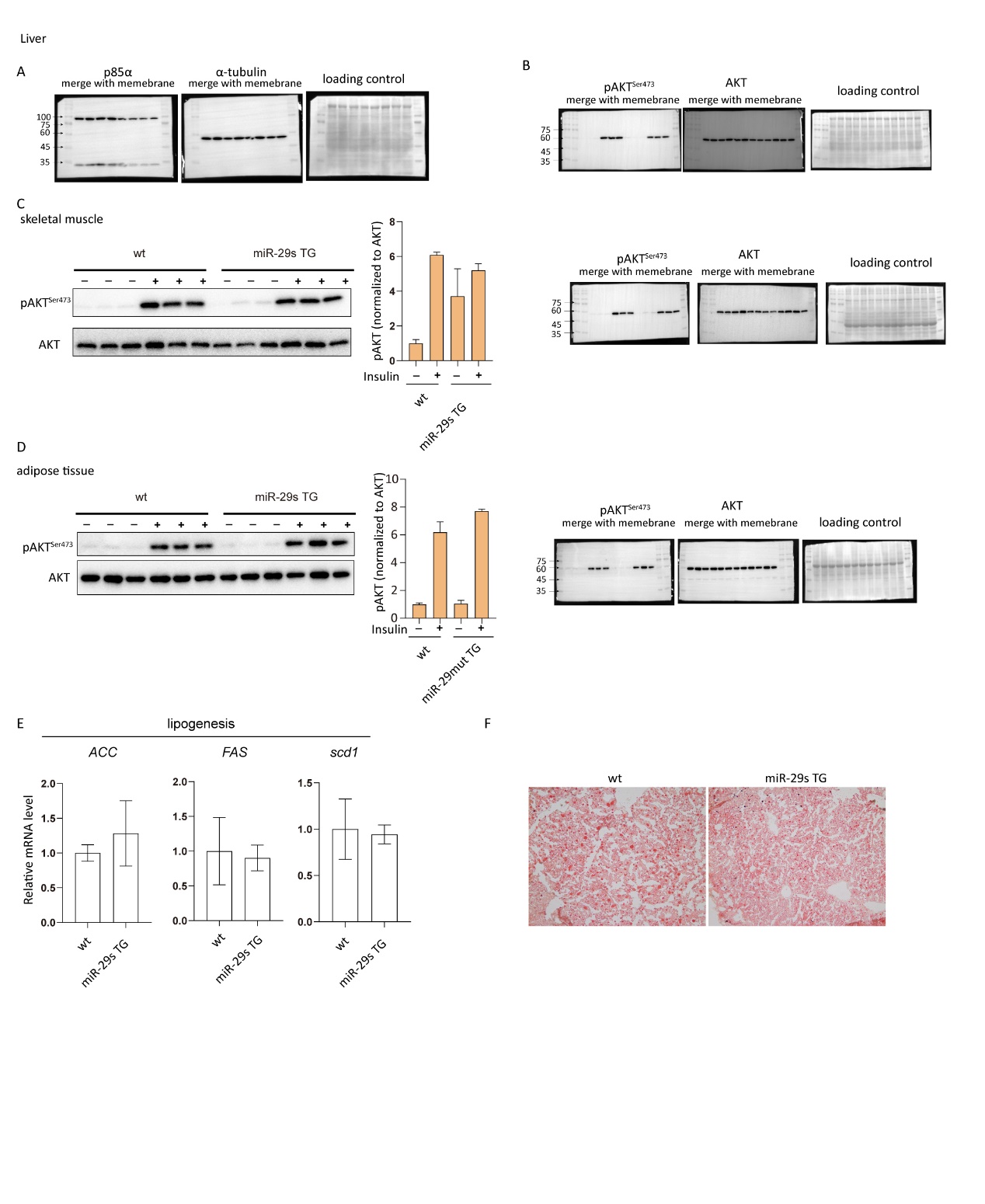


(A-B). The uncropped membrane of p85 α (A) and phosphorylated AKT (C) and their loading controls for figure 4O and 4P. Ponceau staining of the uncropped membrane was used as loading control. The target western blot bands were obtained and merged with the image of uncropped membrane.

(C-D). Representative western blot of insulin-stimulated AKT phosphorylation in the skeletal muscles (C) and gonadal adipose tissues (D) of wile-type or miR-29s TG mice (left panel). Analysis of western blots from n = 3 independent experiments (middle panel). Ponceau staining of the uncropped membrane was used as loading control. The target western blot bands were obtained and merged with the image of uncropped membrane (right panel).

(E). The mRNA levels of lipogenesis genes in livers of wile-type or miR-29s TG mice.

(F). Histochemistry analysis of lipid accumulation in livers of wile-type or miR-29s TG mice.

The liver sections were subjected to H&E staining and Oil-Red-O staining.

Error bars indicate SE (*p < 0.05; **p < 0.01).

Supplementary Figure 7


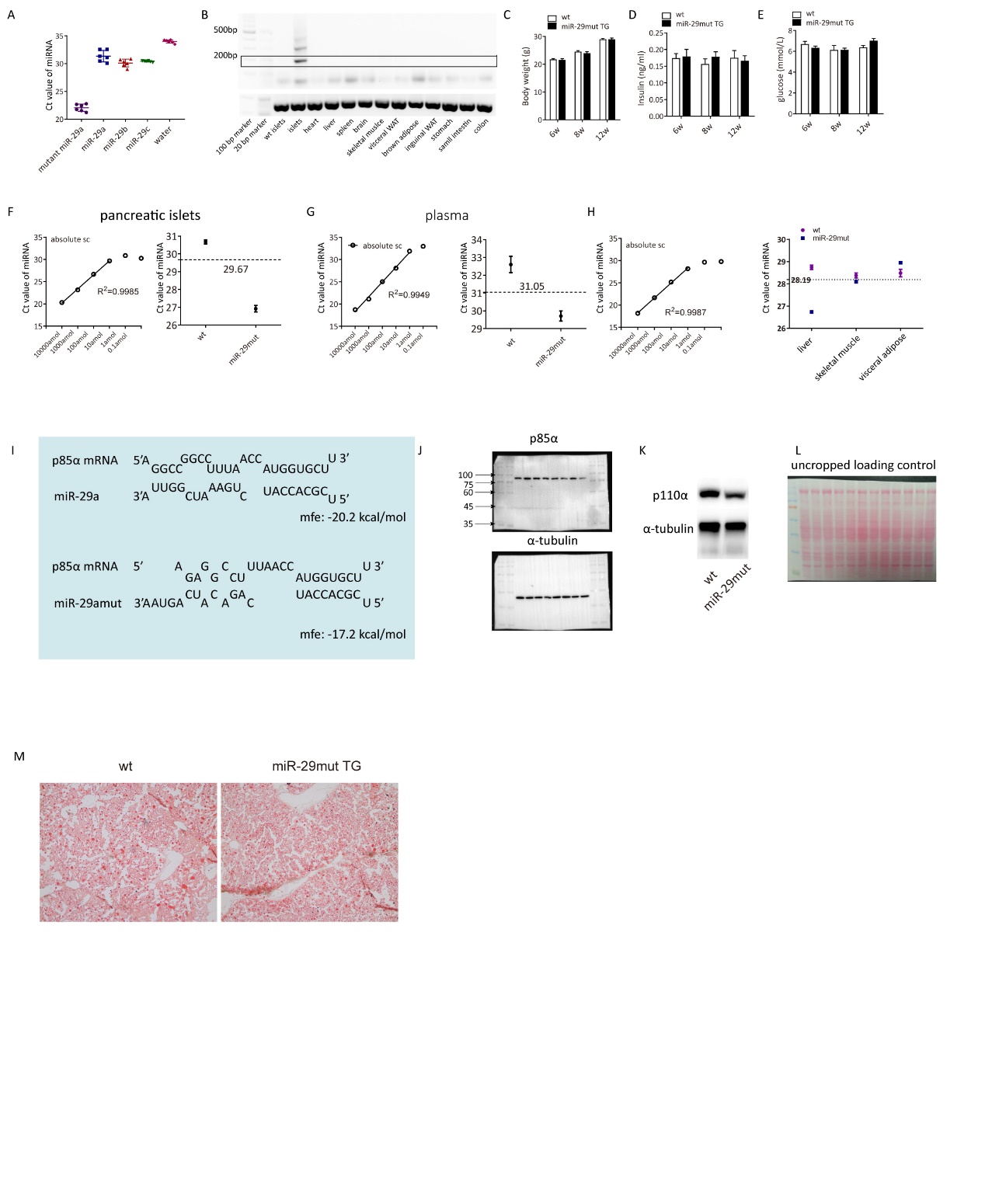


(A). Ct value of mutant miR-29a and endogenous miR-29s analysed by Quantitative real-time PCR using the custom probe for mutant miR-29a. Water was also analysed as negative control.

(B). Semi quantitative RT-PCR analysis of the expression of mutant pre-miR-29a in various tissues.

(C). Body weight of wild-type (chow diet) or miR-29mut TG mice (chow diet) at 6-week, 8-week, and 12-week.

(D). Plasma insulin levels of wild-type or miR-29mut TG mice at 6-week, 8-week, and 12-week assessed by Elisa. Mice were fasted overnight and then subjected to blood extracting.

(E). Blood glucose levels of wild-type or miR-29mut TG mice at 6-week, 8-week, and 12-week. Mice were fasted overnight before test the blood glucose.

(F). Standard curve and ct value of mutant miR-29a in pancreatic islets of wild-type or miR-29mut TG mice analysed by quantitative real-time PCR.

(G). Standard curve and ct value of mutant miR-29a in plasma of wild-type or miR-29mut TG mice analysed by quantitative real-time PCR.

(H). Standard curve and ct value of mutant miR-29a in livers of wild-type or miR-29mut TG mice analysed by quantitative real-time PCR.

(I). Target binding of miR-29a and mutant miR-29a.

(J). The uncropped membrane of p85 α and α-tubulin and loading control for figure 5I.

(K). Representative western blot of p110 and α-tubulin in livers of wild-type or miR-29mut TG mice (left panel). Analysis of p85α western blots from n = 3 independent experiments (right panel).

(L). Ponceau staining of the uncropped membrane in Figure 5J.

(M). Histochemistry analysis of lipid accumulation in livers of wile-type or miR-29mut TG mice.

The liver sections were subjected to H&E staining and Oil-Red-O staining.

Error bars indicate SE (*p < 0.05; **p < 0.01).

Supplementary Figure 8


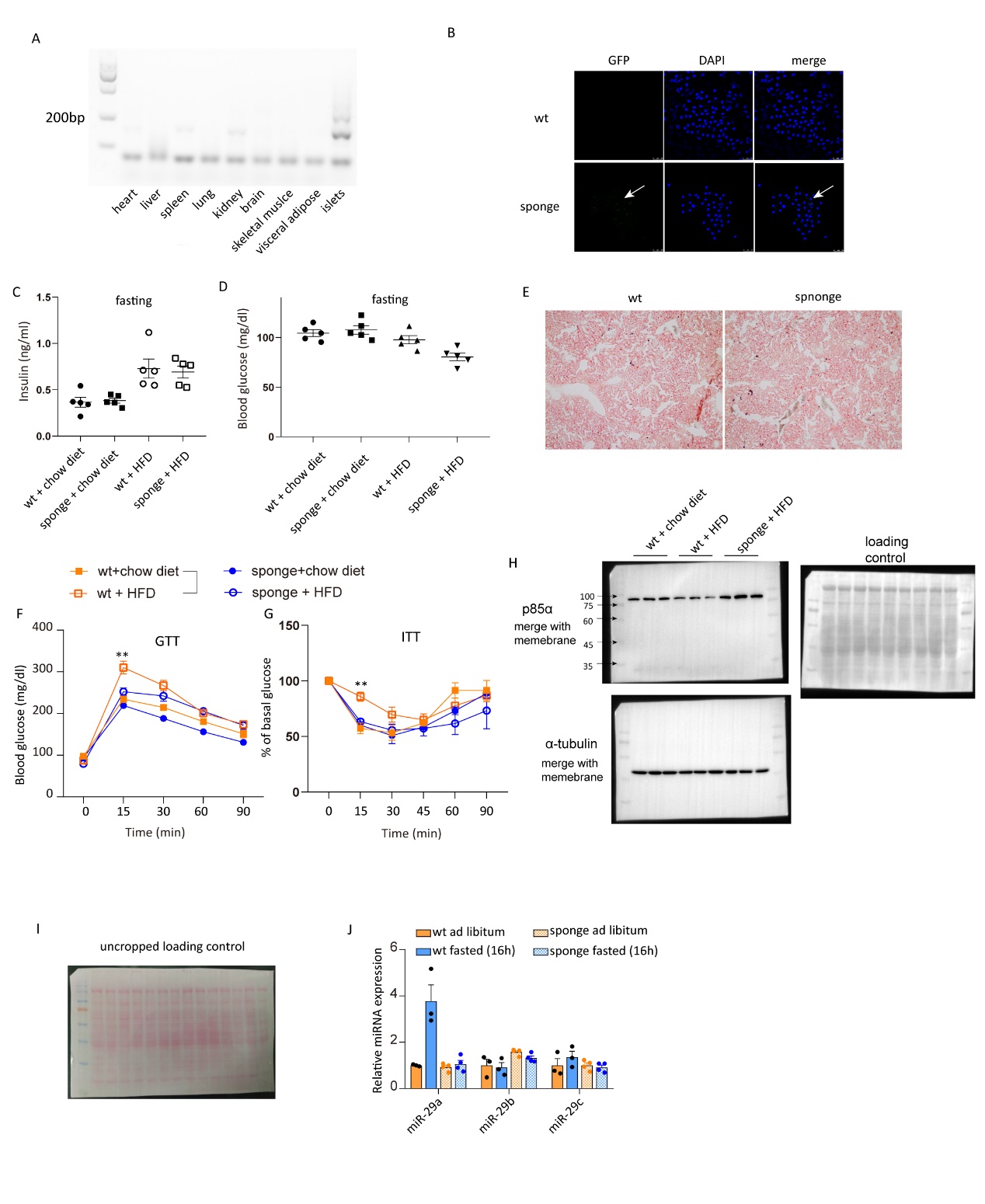


(A). Semi quantitative RT-PCR analysis of GFP expression in various tissues. GFP and sponge construct were both under the control of RIP2 promoter, GFP served as an indicator to test the tissue specific of insert miR-29a/b/c.

(B). Confocal microscopy images of pancreatic islets of wild-type or sponge.

(C). Plasma insulin levels of wild-type and sponge mice on chow diet or HFD respectively as assessed by Elisa.

(D). Blood glucose levels of wild-type and sponge mice on chow diet or HFD respectively. Mice were fasted overnight before testing the blood glucose.

(E). Histochemistry analysis of lipid accumulation in livers of wile-type or sponge mice on chow diet. The liver sections were subjected to H&E staining and Oil-Red-O staining.

(F). Intraperitoneal glucose tolerance test (i.p. GTT) of wild-type and sponge mice on chow diet or HFD respectively (n = 15 for each group).

(G). Insulin tolerance test (ITT) of wild-type and sponge mice on chow diet or HFD respectively (n = 8 for each group).

(H). The uncropped membrane of p85 α and α-tubulin and loading control for Figure 6I.

(I). Ponceau staining of the uncropped membrane in Figure 6J.

(J). qPCR analysis for the miR-29s expression in the plasma of wild-type and sponge mice on chow diet or HFD respectively (n = 3 each group)

Error bars indicate SE (*p < 0.05; **p < 0.01).

Supplementary Figure 9


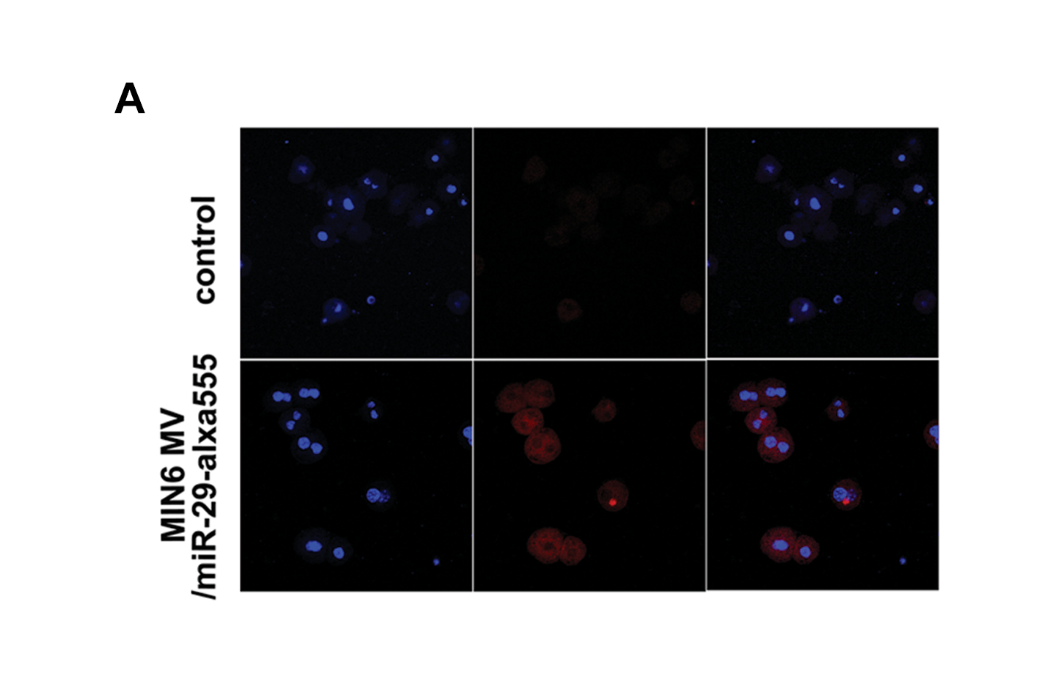


(A). Confocal microscopy images of hepatocytes incubated with normal exosomes or fluorescently-labelled (red) exosomes.
